# NEK2 drives pathogenesis, drug resistance, and LMP1 expression in EBV-positive non-Hodgkin lymphoma

**DOI:** 10.1073/pnas.2535550123

**Published:** 2026-05-14

**Authors:** Maria C. White, Philip T. Lange, Jessica Stewart, Blossom Damania

**Affiliations:** ^a^Lineberger Comprehensive Cancer Center, University of North Carolina at Chapel Hill, Chapel Hill, NC 27599; ^b^ Department of Microbiology and Immunology, University of North Carolina at Chapel Hill, Chapel Hill, NC 27599

**Keywords:** NEK2, EBV, lymphoma, LMP1, drug resistance

## Abstract

Non-Hodgkin lymphoma (NHL) is one of the most common cancers worldwide, representing 90% of malignant lymphomas. NHL is a diverse group of malignancies, and a subset of these lymphomas are caused by infection with the human gammaherpesvirus, Epstein–Barr virus (EBV). Many EBV-positive lymphomas are highly aggressive and rapidly develop resistance to treatment, leading to poor patient outcomes. Here, we identify the cellular kinase, NEK2, as a therapeutic target for EBV-positive NHL. We demonstrate NEK2 protein expression is increased in primary lymphocytes following EBV infection, and that the EBV latency proteins EBNA1, LMP1, and EBNA2 each independently drive NEK2 upregulation. We show NEK2 is necessary for the growth and survival of EBV-positive NHL, and that NEK2 inhibition selectively kills lymphoma cells but not normal lymphocytes. Inhibition of NEK2 resulted in EBV-specific inflammatory cell death characterized by reactive oxygen species accumulation and gasdermin D cleavage. Additionally, expression of the EBV oncoprotein, LMP1, and the cellular oncoprotein, c-myc, were decreased following NEK2 inhibition. Furthermore, we demonstrate multidrug resistance-associated protein 1 (MRP1) is the most active drug resistance transporter protein in EBV-positive NHL. NEK2 inhibition reduced the expression and activity of cellular drug resistance transporter proteins, including MRP1, leading to increased lymphoma cell chemosensitivity. Finally, using a humanized mouse model of EBV-driven lymphomagenesis, we demonstrate NEK2 inhibition significantly decreased tumor burden and tumor incidence while prolonging survival. Taken together, our data suggest NEK2 inhibition as a promising therapeutic strategy for EBV-positive NHL.

## Significance

Epstein–Barr virus (EBV) is associated with certain types of non-Hodgkin lymphoma (NHL). EBV-associated lymphomas are aggressive with limited treatment options, especially for patients whose disease becomes drug-resistant. Here, we characterize inhibition of the cellular protein, NEK2, as a promising therapy for EBV-positive NHL. NEK2 inhibition resulted in lymphoma cell death while simultaneously sensitizing the cells to other cancer treatments. In a humanized mouse model of EBV-associated NHL, NEK2 inhibition significantly prolonged survival and decreased tumor burden, with a subset of animals failing to develop lymphoma. Overall, our data highlight NEK2 as a potential therapeutic target for EBV-positive NHL.

Non-Hodgkin lymphoma (NHL) is one of the most common cancers in both men and women, and global incidence of this malignancy has been increasing over the past few decades (1). NHL is a heterogeneous cancer with many subtypes, some of which are associated with viral infection. Epstein–Barr virus (EBV) is a ubiquitous pathogen, infecting over 90% of the global population. EBV infection is associated with multiple hematologic malignancies, including Burkitt lymphoma (BL), posttransplant lymphoma (PTLD), and NK/T cell lymphomas (NKTL) (2, 3). EBV-positive lymphomas are highly aggressive, and efficacious treatments are often toxic. Additionally, these lymphomas can rapidly develop drug resistance, and few therapeutic options exist for patients whose lymphoma becomes drug resistant (4–6), resulting in unacceptable clinical outcomes. Therefore, more treatment options are needed for EBV-positive NHL.

We previously performed functional cellular kinome profiling to identify kinases with increased activity in both viral and nonviral NHL compared to lymphocytes from healthy donors. One of the kinases that displayed the highest upregulated activity across our NHL panel was Never In Mitosis Gene A related kinase 2, or NEK2 (7). Considering NEK2 was one of only three kinases present in all tested lymphoma cell lines but undetectable in normal B cells, and was highly active in all EBV-positive lymphomas tested, this suggested a potential role for NEK2 in EBV-positive NHL pathogenesis. NEK2 is a serine/threonine kinase that helps control centrosome separation during mitosis, promoting mitotic stability (8–11). High NEK2 expression has been reported in various cancers and has been associated with aggressive disease and poorer patient outcomes (12–14). We and others have recently characterized the pro-oncogenic role of NEK2 in Kaposi’s sarcoma-associated herpesvirus (KSHV)-positive primary effusion lymphoma (PEL) and diffuse large B cell lymphoma, respectively (15, 16). However, an understanding of the mechanistic processes underlying NEK2 activity in lymphoma are still lacking, and no studies to date have examined the role of NEK2 in EBV-positive NHL.

Here, we report NEK2 protein expression is elevated upon primary EBV infection of human B cells and that the EBV latency proteins EBNA1, LMP1, and EBNA2 each independently drive NEK2 upregulation. To test the importance of NEK2 in EBV-positive NHL pathogenesis, we used genetic and pharmacologic inhibition of NEK2 in eight different cell lines encompassing three distinct EBV-associated lymphomas of different cellular origins. We demonstrate NEK2 drives the proliferation of BL (B cell origin), NKTL (natural killer cell and T cell origin), and PTLD (B cell origin), as well as primary human B cells immortalized by EBV infection. We show loss of NEK2 signaling by treating with the NEK2-specific inhibitor, JH295, results in oxidative stress and inflammatory cell death in EBV-positive NHL. Using coisogenic cell lines, we demonstrate that, although both EBV-positive and EBV-negative NHL are susceptible to NEK2 inhibition, EBV-positive cells exhibit significantly more cell death following JH295 treatment compared to their EBV-negative counterparts, and only EBV-positive NHL undergo inflammatory cell death. Additionally, we report inhibition of NEK2 results in progressive loss of the expression of EBV oncoprotein, LMP1, and the cellular oncoprotein, c-myc. Furthermore, we demonstrate JH295 decreases drug resistance in EBV-positive lymphomas and sensitizes the cells to doxorubicin. Using a xenograft mouse model of NKTL, we show JH295 treatment significantly reduces tumor burden. Finally, using a cord blood-humanized mouse model of EBV-driven B cell lymphomagenesis, we demonstrate NEK2 inhibition significantly decreases tumor incidence and burden, resulting in increased overall survival with no negative effects on organ function or the immune system. Overall, our work suggests NEK2 inhibition as a viable treatment strategy for EBV-positive NHL.

## Results

### NEK2 Promotes EBV-Positive NHL Survival.

**Fig. 1.**
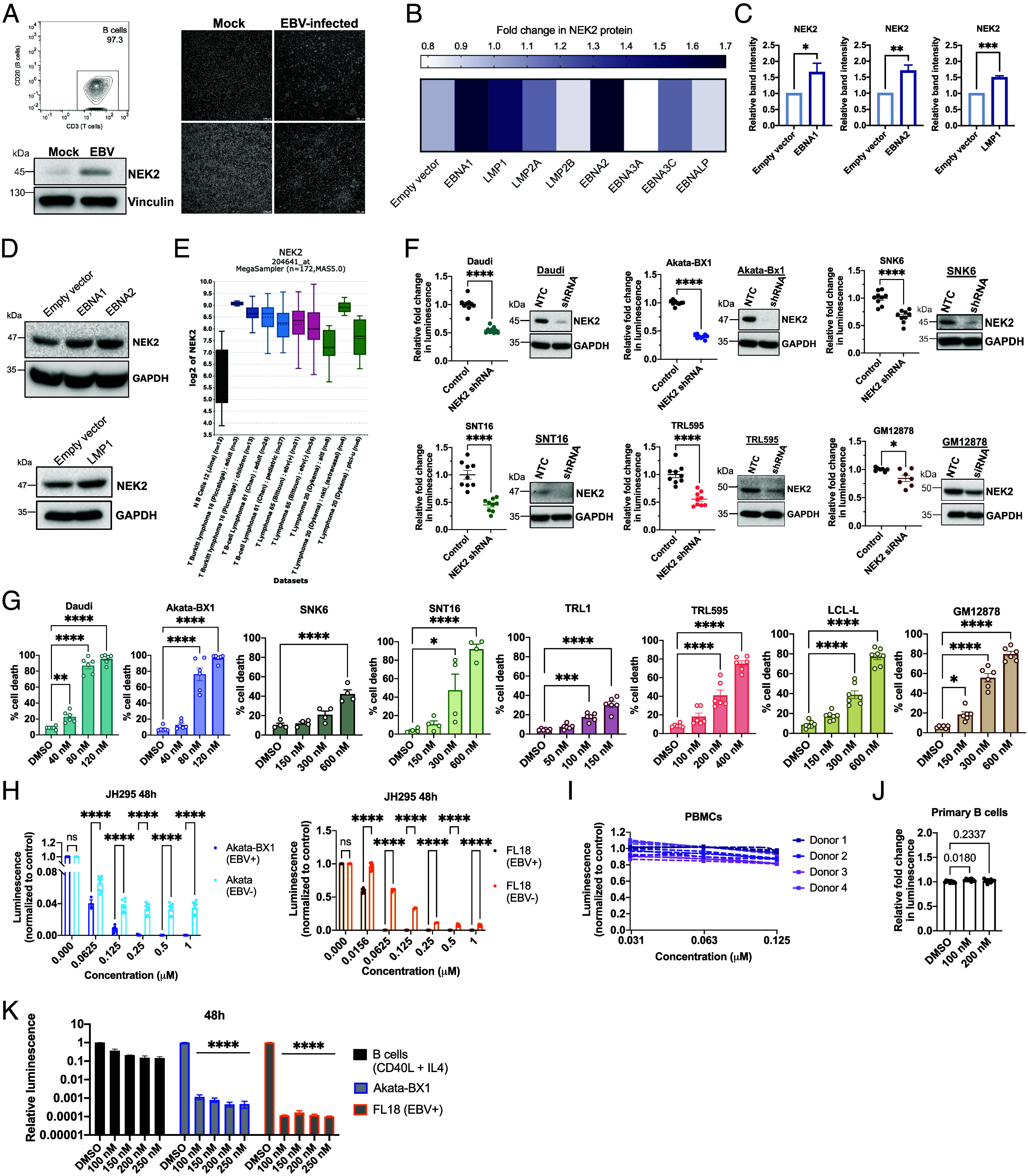
NEK2 expression increases upon primary EBV infection and is necessary for EBV-positive NHL survival. (*A*) Primary B cells were infected ± EBV for 48 h. Cells were imaged to confirm infection (clumping), and NEK2 protein was examined. N = 2 biological replicates. (Scale bar, 100 μm.) (*B*) Heat map of NEK2 protein expression in BJAB cells following EBV open reading frame (ORF) plasmid transfection. N = 2 to 4 biological replicates. Values plotted as means and normalized to the empty vector control set at a fold change of 1.0. (*C* and *D*) NEK2 protein quantification and western blots of NEK2 in EBNA1-, EBNA2-, LMP1-, and control-transfected BJAB cells. NEK2 protein quantification data are normalized to GAPDH, graphed mean ± SEM and analyzed using the *t* test. ***P = 0.0004; **P = 0.0011; *P = 0.0191. N = 2 to 4 biological replicates. (*E*) NEK2 transcripts in normal human tonsillar B cell subsets (black), BL patient tumors (blue), PTLD patient tumors (pink), and T cell—and NKTL patient tumors (green). The middle green bar represents EBV-positive NKTL. (*F*) Viability assays and corresponding immunoblots of EBV-positive NHL with depleted NEK2 protein. All blots except GM12878 (48 h) are 72 h posttransduction. All viability assays except GM12878 (96 h) are 72 h postseeding. Data were normalized to the 0 h luminescence values for each condition and graphed as fold change relative to the nontemplate control. N = 3 biological replicates. Data analyzed using the *t* test and graphed mean ± SEM. ****P < 0.0001; *P < 0.0176. (*G*) Trypan blue assays of cells treated ± JH295 for 48 h. N = 3 to 4 biological replicates. Data analyzed using one-way ANOVA with Dunnett’s multiple comparisons test and graphed mean ± SEM. ****P < 0.0001; ***P = 0.0001; **P = 0.0064; *P = 0.0163 SNT16; *P = 0.0136 GM12878. (*H*) Viability assays of Akata and FL18 coisogenic cell lines treated ± JH295. Data are graphed mean ± SEM and analyzed using two-way ANOVA with Sidak’s multiple comparisons test. ****P < 0.0001. (*I*) Viability assay of peripheral blood mononuclear cells (PBMCs) treated ± JH295. Curves were fit using nonlinear regression and graphed in triplicate. (*J*) Viability assay of B cells treated ± JH295. Data are graphed mean ± SEM and analyzed using one-way ANOVA with Dunnett’s multiple comparisons test (*P*-values on graph). (*K*) Viability assays of stimulated primary B cells (50 ng/mL CD40L and 20 ng/mL IL4 for 72 h) and EBV-positive NHL treated ± JH295. N = 2 biological replicates using two unique donors. Data are graphed mean ± SEM and plotted on a log scale. Data analyzed using two-way ANOVA with Dunnett’s multiple comparisons test. ****P < 0.0001. For (*H***–***K*), data are normalized to the dimethyl sulfoxide (DMSO) control.

First, to determine if primary EBV infection affected NEK2 expression in human lymphocytes, we mock-infected or infected naïve human B cells with EBV and found NEK2 protein levels were elevated in infected cells relative to uninfected cells (Fig. 1*A*). These data suggest EBV infection of B cells induces increased NEK2 expression (*SI Appendix*, Fig. S1*A*). To determine the mechanism of EBV-mediated NEK2 upregulation, we individually transfected EBV-negative BJAB cells with open reading frame plasmids of eight EBV latency proteins (EBNA1, LMP1, LMP2A, LMP2B, EBNA2, EBNA3A, EBNA3C, and EBNALP) and measured NEK2 protein. EBNA1, LMP1, LMP2A, and EBNA3C positively modulate expression of beta-catenin, a protein directly stabilized and controlled by NEK2 (17), while EBNA3A has no reported beta-catenin interaction (18–20). EBNA2 is also a potential regulator of beta-catenin stabilization (21). Results showed independent transfection of EBNA1, EBNA2, and LMP1 plasmids each resulted in a significant increase in NEK2 protein in the EBV-negative cells (Fig. 1 *B*–*D*). To confirm this phenotype, we performed immunoblotting for beta-catenin and observed a significant increase in beta-catenin expression with EBNA1, EBNA2, and LMP1 transfection (*SI Appendix*, Fig. S1 *B* and *C*) but not EBNA3A transfection (*SI Appendix*, Fig. S1*D*). We then examined NEK2 expression in NHL patient tumors using publically available RNA sequencing datasets and found NEK2 transcripts were increased in BL, PTLD, and NKTL relative to B cell subsets from healthy donors (Fig. 1*E*). These patient data corroborate our previous findings of elevated NEK2 expression in both EBV-positive and EBV-negative NHL (7).

Next, to determine if NEK2 was contributing to EBV-positive NHL survival, we depleted NEK2 protein from six EBV-positive NHL cell lines and examined cell viability post-NEK2 depletion. Cell lines used were (details found in *SI Appendix*, Table S1): Daudi and Akata-BX1 (BL); TRL595 (PTLD); SNK6 and SNT16 (NKTL); and GM12878 [lymphoblastoid cell line (LCL) generated by transforming primary B cells with EBV]. We found NEK2 depletion significantly reduced viability in all cell lines (Fig. 1*F*), suggesting NEK2 is important for EBV-positive NHL survival. To determine if blocking NEK2 activity was sufficient to induce cell death, we used the irreversible and highly NEK2-specific inhibitor, JH295 (22), to inhibit NEK2 signaling in eight EBV-positive NHL cell lines (the above six lines plus an additional PTLD cell line, TRL1, and an additional LCL, LCL-L). We found JH295 treatment induced cell death at submicromolar concentrations in all cell lines as measured by trypan blue assay (Fig. 1*G*), cell viability assay (*SI Appendix*, Fig. S1*E*), and PI staining (*SI Appendix*, Fig. S1*F*). We also observed a decrease in EBV-positive NHL proliferation with JH295 as measured by live cell counts (*SI Appendix*, Fig. S1*G*) and GI50 determination (*SI Appendix*, Table S2). To confirm our findings, we compared the JH295 GI50 values to those of another irreversible NEK2-specific inhibitor, NBI-961. Results showed similar GI50 values for both JH295 and NBI-961 in EBV-positive NHL (*SI Appendix*, Table S3). We also tested a reversible and less specific (compared to JH295 and NBI-961) NEK2 inhibitor, NCL 00017509, and found the cells were resistant to this drug up to the highest tested concentration of 8 μM (*SI Appendix*, Fig. S1*H*), further suggesting JH295-mediated cell death was NEK2-specific and the result of irreversible kinase inhibition. Finally, using RNAi data from the DepMap Portal we found lymphoma cell lines have a dependency on NEK2, further validating our approach (*SI Appendix*, Fig. S1*I*).

After observing robust cell death in EBV-positive NHL following NEK2 inhibition, we determined the extent to which EBV infection was driving this phenotype. We used two pairs of coisogenic cell lines, the BL cell line, Akata, and the follicular lymphoma cell line, FL18, which were either positive or negative for EBV. We treated these coisogenic cell line pairs with JH295 and measured cell death. The EBV-positive cells displayed significantly more death than their EBV-negative counterparts (Fig. 1*H*), suggesting EBV infection renders NHL more susceptible to NEK2 inhibition. Additionally, we treated the EBV-negative BL cell line, BJAB, with JH295 and found it was more resistant compared to the EBV-positive BL cell line, Akata-BX1 (*SI Appendix*, Fig. S1*J*). Next, to test if NEK2 inhibition impacted normal cell viability, we treated PBMCs from four unique donors with JH295 and observed no significant cell death (Fig. 1*I*). We then isolated primary B cells from healthy donor PBMCs (*SI Appendix*, Fig. S1*K*) and treated them with JH295. Similarly, no cell death was observed in the naïve B cells (Fig. 1*J*). To test the effects of NEK2 inhibition on rapidly dividing primary cells, we stimulated primary B cells with CD40L and IL4, which resulted in significant proliferation (*SI Appendix*, Fig. S1*L*). We treated these proliferating B cells with JH295 alongside the EBV-positive Akata-BX1 and EBV-positive FL18 cell lines and measured cell viability. Importantly, the rapidly dividing healthy B lymphocytes were magnitudes less susceptible to JH295 compared to EBV-positive B cell NHL (Fig. 1*K*), suggesting inhibition of NEK2 preferentially causes lymphoma cell death.

### NEK2 Inhibition Results in Hydrogen Peroxide (H_2_O_2_) Accumulation and EBV-Positive NHL Inflammatory Cell Death.

**Fig. 2.**
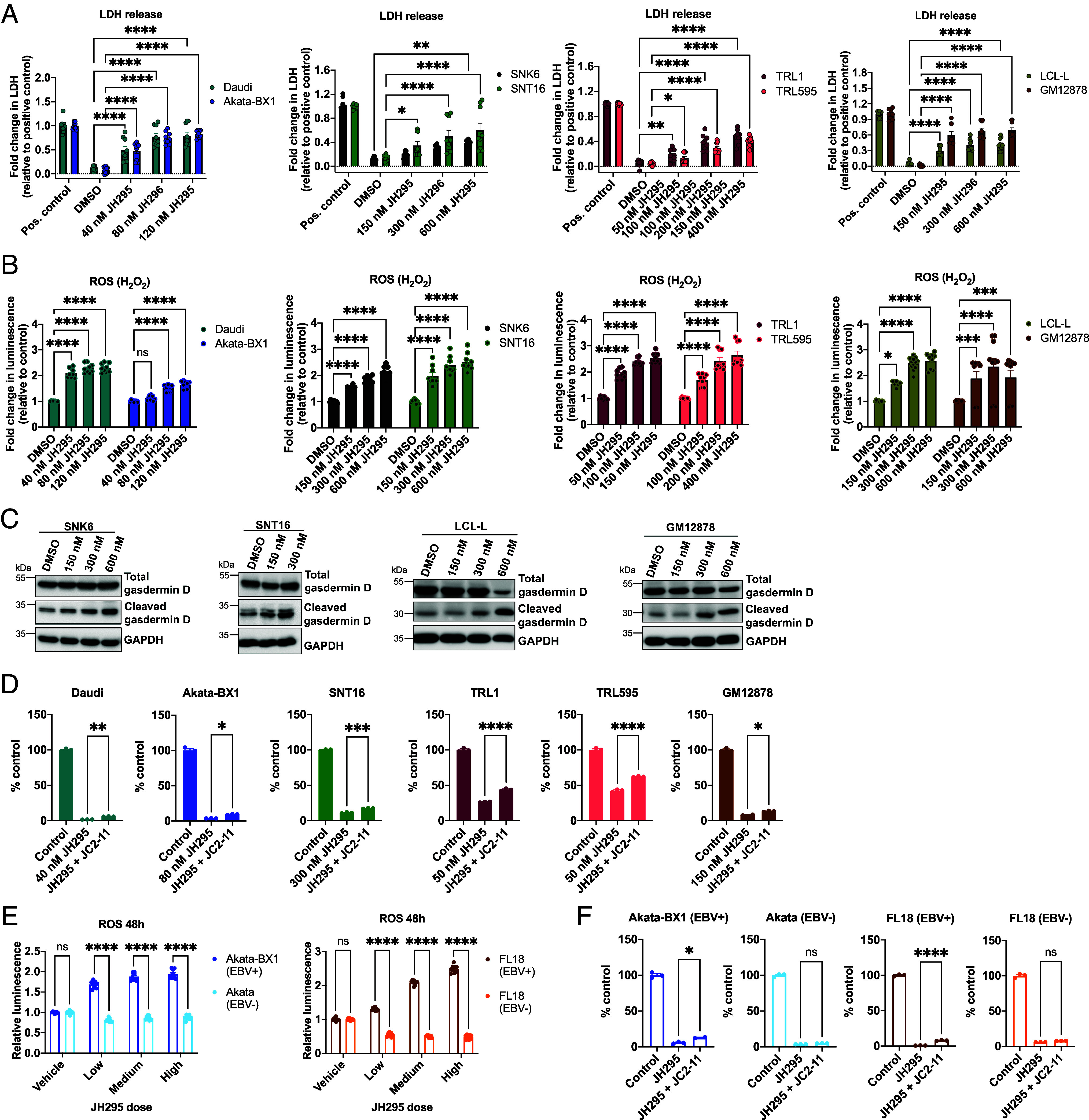
EBV-positive NHL undergo inflammatory cell death following NEK2 inhibition. (*A*) LDH release assays of cells treated ± JH295 for 4 h. N = 3 biological replicates. Data graphed as fold change relative to the positive control (maximum LDH release by cell lysis), analyzed using two-way ANOVA with Sidak’s multiple comparisons test, and graphed mean ± SEM. ****P < 0.0001; **P = 0.0044 SNK6; **P = 0.0034 TRL1; *P = 0.0363 SNT16; *P = 0.0437 TRL595. (*B*) ROS assays of EBV-positive NHL treated ± JH295 for 48 h. N = 2 to 4 biological replicates. Data graphed as fold change in luminescence relative to the DMSO control. Data analyzed using two-way ANOVA with Holm–Sidak’s multiple comparisons test and graphed mean ± SEM. ****P < 0.0001; ***P = 0.0001; *P = 0.0208. (*C*) Western blot of gasdermin D in cells treated ± JH295 for 48 h. N = 2 to 3 biological replicates. (*D*) Viability assays of EBV-positive NHL treated with control (DMSO), JH295, or JH295 plus 1.5 μM JC2-11 for 24 h. Data graphed as percent control (i.e., DMSO was set to 100% luminescence). Data are representative of N = 2 to 3 biological replicates, analyzed using one-way ANOVA with Dunnett’s multiple comparisons test, and graphed mean + SEM. ****P < 0.0001; ***P = 0.0002; **P = 0.0049; *P < 0.05. (*E*) ROS assays of Akata and FL18 coisogenic cell lines treated ± JH295 for 48 h. N = 3 biological replicates. Data graphed as fold change in luminescence relative to the DMSO control. Data analyzed using two-way ANOVA with Holm-Sidak‘s multiple comparisons test and graphed as mean ± SEM. ****P < 0.0001. JH295 concentrations used: Akata-BX1 and Akata low (62 nM), medium (90 nM), and high (125 nM); FL18+ low (16 nM), medium (31 nM), and high (62 nM); and FL18− low (125 nM), medium (250 nM), and high (500 nM). (*F*) Viability assays of Akata and FL18 coisogenic cell lines treated with DMSO, JH295, or JH295 plus 1.5 μM JC2-11 for 24 h. Data graphed as percent control. N = 3 biological replicates. Data analyzed using one-way ANOVA with Dunnett’s multiple comparisons test, and graphed mean + SEM. ****P < 0.0001; *P < 0.05. JH295 concentrations used: Daudi = 40 nM; Akata-BX1 = 80 nM; SNT16 = 300 nM; TRL1 and TRL595 = 50 nM; GM12878 = 150 nM; coisogenic Akata cell lines = 125 nM; FL18 EBV-positive = 62 nM; FL18 EBV-negative = 500 nM. SNK6, LCL-L, and GM12878 loading controls (GAPDH) in *C* and *SI Appendix*, Fig. S4*C* are the same.

We then determined the mechanism by which the EBV-positive NHL cells were dying following JH295 treatment. As NEK2 is a mitotic kinase, we first tested if NEK2 inhibition led to cell cycle arrest. Following JH295 treatment, we quantified the percentage of cells in each cell cycle phase and observed slight but significant cell cycle arrest in 7/8 cell lines (*SI Appendix*, Fig. S2 *A*–*D*). Some cell lines arrested in G1 phase (NKTL and LCL) while others arrested in G2/M phase (BL and TRL595), suggesting the phase of cell cycle arrest following NEK2 inhibition is lymphoma subtype-dependent. We also quantified sub-G1 content, as high sub-G1 content can be indicative of apoptotic cell death. We found slight sub-G1 content elevation in 7/8 cell lines following NEK2 inhibition (*SI Appendix*, Fig. S2*E*). The relatively low magnitude of sub-G1 content, together with no observed increase in cleaved caspase 3 or cleaved PARP protein expression, suggested the cells were dying by a mechanism other than apoptosis. To confirm this, we performed lactate dehydrogenase (LDH) assays 4 h after JH295 treatment on EBV-positive NHL and the KSHV-associated PEL cell line, JSC1. We previously reported multiple PEL cell lines, including JSC1, undergo apoptotic cell death following JH295 treatment (15). We found NEK2 inhibition induced significant LDH release in all EBV-positive NHL cells, but not the JSC1 cells (Fig. 2*A* and *SI Appendix*, Fig. S3*A*). These data suggested EBV-positive NHL were not undergoing apoptosis, but rather non-apoptotic cell death that quickly compromised the plasma membrane. To elucidate these findings, we quantified levels of the reactive oxygen species (ROS), H_2_O_2_, in EBV-positive NHL following JH295 treatment, as a recent study demonstrated NEK2 inhibition facilitates ROS production to induce non-apoptotic cell death in gastric cancer (23). We observed significantly elevated H_2_O_2_ levels in all cell lines following NEK2 inhibition (Fig. 2*B*). In cancer, H_2_O_2_ can be scavenged to promote cell survival, and one described scavenger of H_2_O_2_ is lactate (24). Thus, we next measured lactate abundancy and found lactate levels were significantly decreased in all cell lines following NEK2 inhibition (*SI Appendix*, Fig. S3*B*). Additionally, we found cleaved gasdermin D protein was elevated following JH295 treatment (Fig. 2*C*). Early plasma membrane deterioration, LDH release, decreased lactate, H_2_O_2_ accumulation, and elevated cleaved gasdermin D can all be indicative of inflammatory cell death (25–28). To confirm this, we treated the cells with JH295 in combination with JC2-11, a drug that blocks inflammatory mediators such as LDH release, ROS production, and gasdermin D cleavage, all of which were observed in EBV-positive NHL following JH295 treatment. We found JC2-11 partially rescued JH295-mediated cell death (Fig. 2*D* and *SI Appendix*, Fig. S3 *C* and *D*), further suggesting NEK2 inhibition was inducing inflammatory cell death in EBV-positive NHL. To determine if inflammatory cell death was specific to EBV infection, we measured ROS production in the coisogenic EBV-positive and EBV-negative Akata and FL18 cell lines following NEK2 inhibition. Interestingly, only the EBV-positive cells accumulated H_2_O_2_ in response to JH295 (Fig. 2*E*). When we treated these coisogenic cell lines with JH295 in combination with JC2-11, cell death was partially rescued only in the EBV-positive cells (Fig. 2*F* and *SI Appendix*, Fig. S3 *E* and *F*). Overall, these data suggest EBV-positive NHL, but not EBV-negative NHL, undergo inflammatory cell death following NEK2 inhibition.

### Inhibition of NEK2 Decreases Expression of Pro-Survival Cellular Proteins and the EBV Oncoprotein, LMP1.

**Fig. 3.**
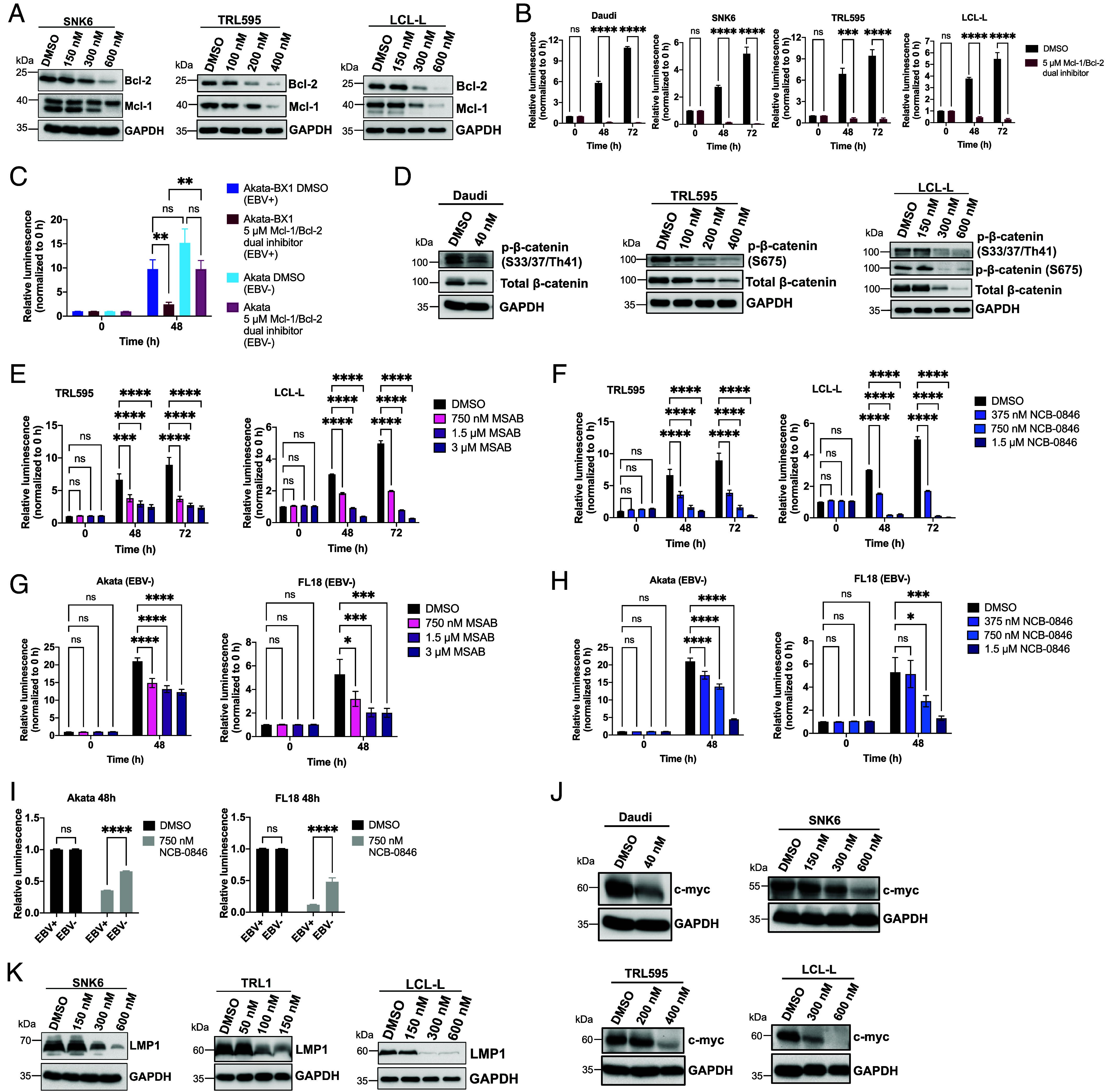
JH295 decreases expression of cellular pro-survival proteins and the EBV oncoprotein, LMP1. (*A*) Western blot of Mcl-1 and Bcl-2 in EBV-positive NHL treated ± JH295 for 48 h. N = 3 biological replicates. (*B* and *C*) Viability assays of cells treated with a Mcl-1/Bcl-2 dual inhibitor. N = 3 biological replicates. Data analyzed using two-way ANOVA with Sidak’s (*B*) or Tukey’s (*C*) multiple comparisons test and graphed mean ± SEM. ****P < 0.0001; ***P = 0.0001; **P < 0.01. (*D*) Western blot of phosphorylated and total beta-catenin in cells treated ± JH295 for 48 h. N = 3 biological replicates. The blot for phosphorylated beta-catenin was stripped and reprobed with antibody against total beta-catenin. (*E*) Viability assays of EBV-positive NHL treated with a beta-catenin inhibitor. N = 2 to 3 biological replicates. Data analyzed using two-way ANOVA with Dunnett’s multiple comparisons test and graphed mean ± SEM. ****P < 0.0001; ***P = 0.0005. (*F*) Viability assays of EBV-positive NHL treated with a beta-catenin transcription inhibitor. N = 2 to 3 biological replicates. Data analyzed using two-way ANOVA with Dunnett’s multiple comparisons test and graphed mean ± SEM. ****P < 0.0001. (*G*) Viability assays of EBV-negative NHL treated with a beta-catenin inhibitor. N = 3 biological replicates. Data analyzed using two-way ANOVA with Dunnett‘s multiple comparisons test and graphed mean ± SEM. ****P < 0.0001; ***P = 0.0003; *P = 0.0239. (*H*) Viability assays of EBV-negative NHL treated with a beta-catenin transcription inhibitor. N = 3 biological replicates. Data analyzed using two-way ANOVA with Dunnett’s multiple comparisons test and graphed mean ± SEM. ****P < 0.0001; ***P = 0.0002; *P = 0.0231. (*I*) Viability assays of EBV-positive and EBV-negative coisogenic NHL cell lines treated with NCB-0846 for 48 h. N = 2 to 3 biological replicates. Data analyzed using two-way ANOVA with Sidak’s multiple comparisons test and graphed mean ± SEM. ****P < 0.0001. (*J*) Western blot of c-myc in cells treated ± JH295 for 48 h. N = 3 biological replicates. (*K*) Western blot of LMP1 in cells treated ± JH295 for 48 h. N = 3 biological replicates. Daudi loading controls (GAPDH) in *D*, *J*, and *SI Appendix*, Fig. S4 *A* and *C* are the same. SNK6 loading controls in *A*, *K*, and Fig. 4*A* are the same. TRL595 loading controls in *D*, Fig. 4*A*, and *SI Appendix*, Fig. S4*C* are the same. LCL-L loading controls in *A*, *D*, and *K* are the same. For viability assays, data were normalized to the 0 h luminescence values for each condition.

To further understand the underlying mechanism(s) governing JH295-mediated cell death in EBV-positive NHL, we examined protein expression following JH295 treatment and found decreased levels of the pro-survival proteins, Bcl-2 and Mcl-1, in all cell lines (Fig. 3*A* and *SI Appendix*, Fig. S4*A*). When both Bcl-2 and Mcl-1 were simultaneously inhibited using a dual drug inhibitor, we also observed cell death (Fig. 3*B* and *SI Appendix*, Fig. S4*B*). Interestingly, while both EBV-positive Akata-BX1 cells and EBV-negative Akata cells were susceptible to Mcl-1/Bcl-2 dual inhibition, the EBV-positive Akata-BX1 cells displayed significantly more cell death than their EBV-negative counterpart (Fig. 3*C*). We also observed a decrease in Bcl-xL protein expression with JH295 (*SI Appendix*, Fig. S4*C*), and treatment with a Bcl-xL inhibitor also led to cell death (*SI Appendix*, Fig. S4*D*). Together, these data suggest the pro-survival proteins Bcl-2, Mcl-1, and Bcl-xL are modulated by NEK2 to promote EBV-positive NHL survival, even in the absence of apoptosis. Additionally, we observed a decrease in the phosphorylation and expression of the NEK2-interacting protein and transcription factor, beta-catenin, in the EBV-positive NHL cell lines (Fig. 3*D* and *SI Appendix*, Fig. S4*E*). Beta-catenin is a direct target of NEK2, and NEK2 phosphorylates beta-catenin at the S675 and S33/37/Th41 sites examined (Fig. 3*D* and *SI Appendix*, Fig. S4*E*). When we independently inhibited beta-catenin activity (Fig. 3*E* and *SI Appendix*, Fig. S4*F*) and transcription of beta-catenin target genes (Fig. 3*F* and *SI Appendix*, Fig. S4*G*), we found both inhibitors induced cell death, suggesting both beta-catenin and its downstream effectors contribute to EBV-positive NHL survival. We also tested the effects of beta-catenin inhibition on EBV-negative NHL and observed similar results (Fig. 3 *G* and *H* and *SI Appendix*, Fig. S4*H*). Interestingly, however, EBV infection resulted in significantly more cell death following transcriptional inhibition of beta-catenin target genes (Fig. 3*I*). These data suggest expression of proteins controlled by the beta-catenin transcriptional complex are more important for EBV-positive NHL survival. The cellular oncogene, c-myc, is one protein transcriptionally regulated by beta-catenin. Additionally, BL pathogenesis is driven by a c-myc translocation, independent of beta-catenin signaling. We observed decreased expression of c-myc following NEK2 inhibition in EBV-positive NHL, including BL (Fig. 3*J* and *SI Appendix*, Fig. S4*I*). Finally, we examined multiple EBV proteins following NEK2 inhibition and found expression of the EBV oncoprotein, LMP1, was decreased in all cell lines that express this protein (BL do not express LMP1) (Fig. 3*K* and *SI Appendix*, Fig. S4*J*), while expression of EBNA1, an EBV latency protein common to all cell lines, remained unchanged (*SI Appendix*, Fig. S4*K*). EBV-transformed B cells with depleted LMP1 expression do not proliferate (29), highlighting the essential role of LMP1 for EBV-infected cell growth. Taken together, these data suggest NEK2 modulates cellular and viral protein expression to support EBV-positive NHL survival.

### JH295 Decreases Multidrug Resistance-Associated Protein 1 (MRP1)-Mediated Drug Resistance in EBV-Positive NHL.

**Fig. 4.**
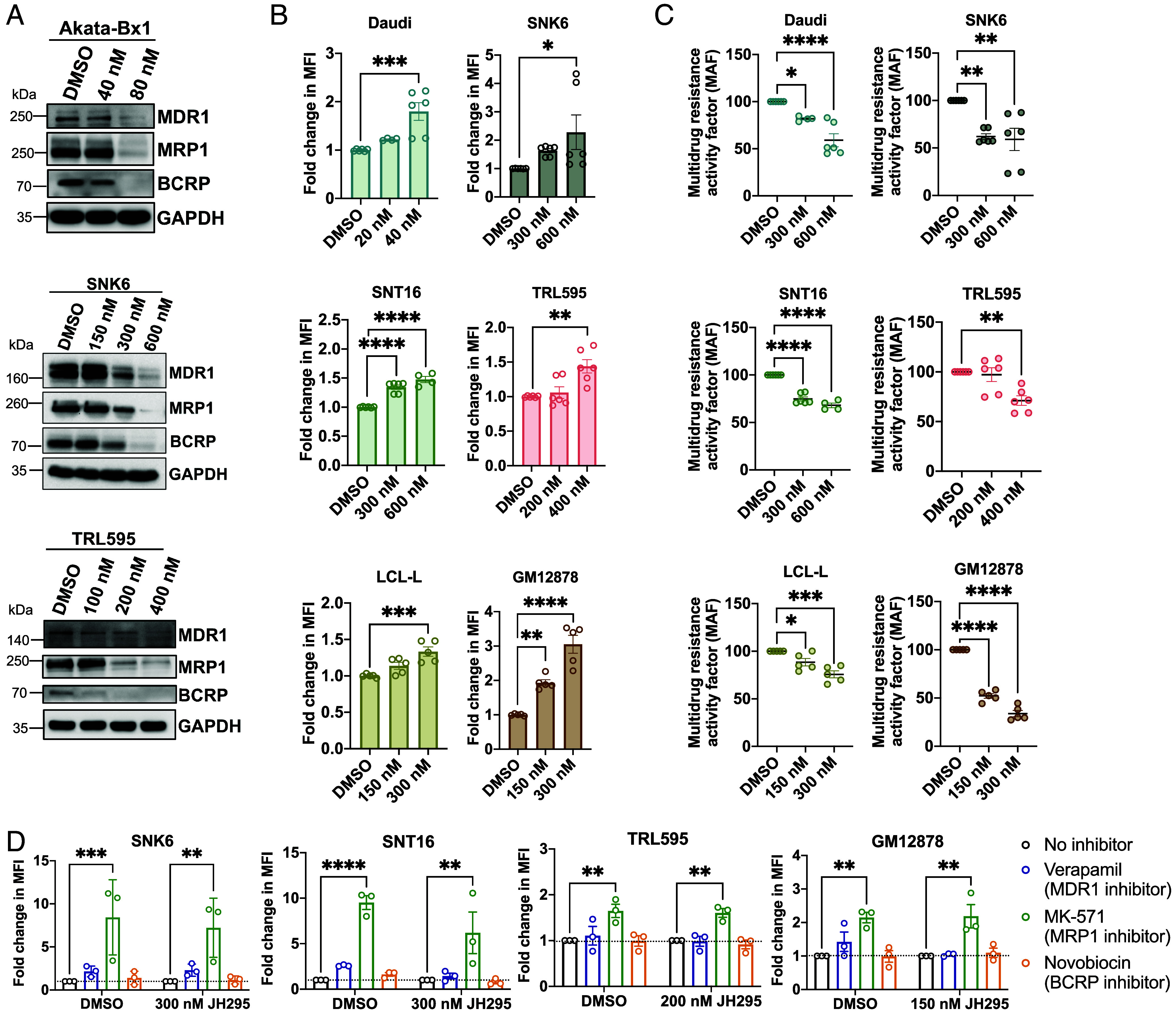
NEK2 inhibition reduces MRP1-mediated drug resistance in EBV-positive NHL. (*A*) Western blot of MDR1, MRP1, and BCRP in cells treated ± JH295 for 48 h. N = 3 biological replicates. The SNK6 blot for MDR1 was stripped and reprobed with antibody against MRP1. (*B*) ABC transporter activity in cells treated ± JH295 for 48 h. Cells were loaded with DiOC_2_(3) and substrate remaining inside the cells after an efflux period was quantified. Data graphed as fold change in mean fluorescence intensity (MFI) relative to the control. N = 3 biological replicates. Data analyzed using one-way ANOVA with Dunnett’s multiple comparisons test and graphed mean ± SEM. ****P < 0.0001; ***P = 0.0005 Daudi; ***P = 0.0006 LCL-L; **P = 0.0015 TRL595; **P = 0.0024 GM12878; *P = 0.0394. (*C*) Multidrug resistance activity factor (MAF) calculations using the data in (*B*). Data are normalized to the control (set at a MAF of 100). Data analyzed using one-way ANOVA with Dunnett’s multiple comparisons test and graphed mean ± SEM. ****P < 0.0001; ***P = 0.0003; **P < 0.002; *P = 0.0361 Daudi; *P = 0.0429 LCL-L. (*D*) eFLUXX-ID® Green assays measuring activity of each ABC transporter in cells treated ± JH295 for 48 h. Data graphed as fold change in MFI relative to the no-inhibitor control for each condition. Dotted line represents no change (y = 1). N = 3 biological replicates. Data analyzed using two-way ANOVA with Dunnett’s multiple comparisons test and graphed mean ± SEM. ****P < 0.0001; ***P < 0.0005; **P ≤ 0.005. SNK6 loading controls (GAPDH) in *A*, Fig. 3*A*, and Fig. 3*K* are the same. TRL595 loading controls in *A*, Fig. 3*D*, and *SI Appendix*, Fig. S4*C* are the same.

Considering the propensity of EBV-positive NHL to become drug resistant and the reported role of NEK2 in facilitating cancer drug resistance (12), we tested if JH295 affected the expression and/or function of proteins involved in cellular drug resistance pathways. Multidrug resistance 1 (MDR1), MRP1, and breast cancer resistance protein (BCRP) belong to the adenosine triphosphate binding cassette (ABC) transporter protein family and are the most common mediators of drug resistance in cancer (30). Normally, ABC transporters remove toxins and other harmful substrates from cells, helping maintain cellular homeostasis (31). However, under malignant conditions, ABC transporters can also remove anticancer drugs, negating their effect and facilitating resistance (32). Notably, MDR1, MRP1, and BCRP can all be regulated by the beta-catenin signaling pathway (33–35). We treated EBV-positive NHL with JH295 and performed immunoblotting for MDR1, MRP1, and BCRP. Strikingly, we observed a dose-dependent decrease in expression of all three ABC transporters in all cell lines with JH295 (Fig. 4*A* and *SI Appendix*, Fig. S5*A*). To test if ABC transporter inhibition alone was enough to induce cell death, we treated the cells with individual drug inhibitors of each transporter (MDR1 = verapamil; MRP1 = MK571; BCRP = novobiocin) and measured cell viability. With the exception of BCRP inhibition in TRL1 cells, single inhibition of each inhibitor induced cell death (*SI Appendix*, Fig. S5*B*), suggesting function of these transporters contributes to lymphoma cell viability. To measure if JH295 itself reduced ABC transporter activity, we used a multidrug resistance assay in which a fluorescent molecule transported by ABC transporters, DiOC_2_(3), is loaded into the cells and the efflux (cellular exit) of the substrate is measured by flow cytometry [as described in (15)]. The substrate remaining inside the cells after the efflux period is quantified, so substrate retention inversely correlates to transporter activity. JH295 significantly increased the relative amount of substrate retained within the cells after the efflux period in a dose-dependent manner (Fig. 4*B* and *SI Appendix*, Fig. S5*C*), suggesting NEK2 inhibition decreases ABC transporter activity. These data were then used to calculate the multidrug resistance activity factor (MAF) under each treatment condition. The MAF significantly decreased in all cell lines following NEK2 inhibition (Fig. 4*C* and *SI Appendix*, Fig. S5*D*). Altogether, these data suggest inhibition of NEK2 reduces drug resistance activity in EBV-positive NHL.

Currently, it is unclear which ABC transporter(s) mediate drug resistance in EBV-positive NHL. To address this question, we quantified the individual contributions of MDR1, MRP1, and BCRP to substrate efflux using the eFLUXX-ID® Green assay. This assay measures efflux of a proprietary fluorescent substrate through all ABC transporters simultaneously as well as through each transporter independently. We found MRP1 was the most active transporter, with MDR1 having slight activity and BCRP having little to no activity (Fig. 4*D* and *SI Appendix*, Fig. S5*E*). This phenotype was the most striking in NKTL, where MRP1 inhibition almost entirely blocked cellular substrate efflux (*SI Appendix*, Fig. S5*F*). MRP1-dependent efflux was present with and without JH295 (Fig. 4*D* and *SI Appendix*, Fig. S5*E*), indicating high MRP1 activity is inherent to EBV-positive NHL. Overall, these data suggest MRP1 mediates ABC transporter protein-dependent drug resistance in EBV-positive NHL.

### JH295 Does Not Decrease Protein Expression in Nonmalignant Cells and Does Not Induce Global Protein Downregulation in Malignant Cells.

We found NEK2 inhibition in EBV-positive NHL resulted in decreased expression of cellular proteins including Bcl-2, Mcl-1, Bcl-xL, beta-catenin, and ABC transporters. To ensure JH295 did not suppress protein expression in normal cells, we performed immunoblotting for these proteins on JH295-treated PBMCs. We found none of the proteins were decreased in PBMCs following NEK2 inhibition (*SI Appendix*, Fig. S6*A*), suggesting JH295-mediated protein modulation is specific to malignant cells. To ensure JH295 did not induce global downregulation of EBV-positive NHL protein expression, we performed protein staining (Ponceau S) on an immunoblot containing protein lysate from EBV-positive NHL treated with and without JH295. Results showed global protein expression was similar across each cell line regardless of drug treatment (*SI Appendix*, Fig. S6*B*), suggesting JH295-mediated protein modulation is targeted and not a global reduction due to cell death.

### NEK2 Inhibition Decreases EBV-Positive Lymphoma Burden In Vivo.

**Fig. 5.**
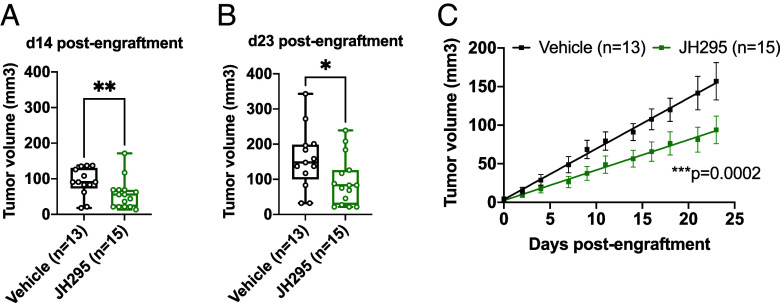
JH295 reduces tumor burden in a SNK6 xenograft mouse model. (*A* and *B*) SNK6 tumor volume in mice treated with DMSO or 15 mg/kg JH295 at day 14 (*A*) and day 23 (*B*) postengraftment. N = 3 biological replicates. Data plotted min-to-max and analyzed using the Mann–Whitney test. **P = 0.0098; *P = 0.0177. (*C*) Linear regression of SNK6 tumor growth. Means were plotted and the difference between slopes was calculated (*P*-value on the graph). N = 3 biological replicates.

Next, we sought to investigate the therapeutic potential of our in vitro findings using mouse models of EBV-positive lymphoma. First, we determined the efficacy of NEK2 inhibition in NKTL using a xenograft mouse model, in which 1 million SNK6 cells were subcutaneously injected into NOD scid gamma (NSG) mice. 4 d after palpable tumors had formed (d0 postengraftment), mice were randomized by tumor burden and treated with DMSO or 15 mg/kg JH295 until d23 postengraftment. JH295 had no effect on the weights of the mice (*SI Appendix*, Fig. S7*A*), and the tumor volumes at d0 were similar in each treatment group (*SI Appendix*, Fig. S7*B*). However, tumor volumes were significantly lower in the JH295-treated mice at d14 (Fig. 5*A*) and d23 (Fig. 5*B*) postengraftment compared to control mice, suggesting NEK2 inhibition decreases NKTL tumor burden in vivo. Finally, linear regression analysis revealed the rate of SNK6 tumor growth in the JH295-treated mice was lower than that of control mice (Fig. 5*C*), suggesting JH295 treatment delays NKTL progression.

**Fig. 6.**
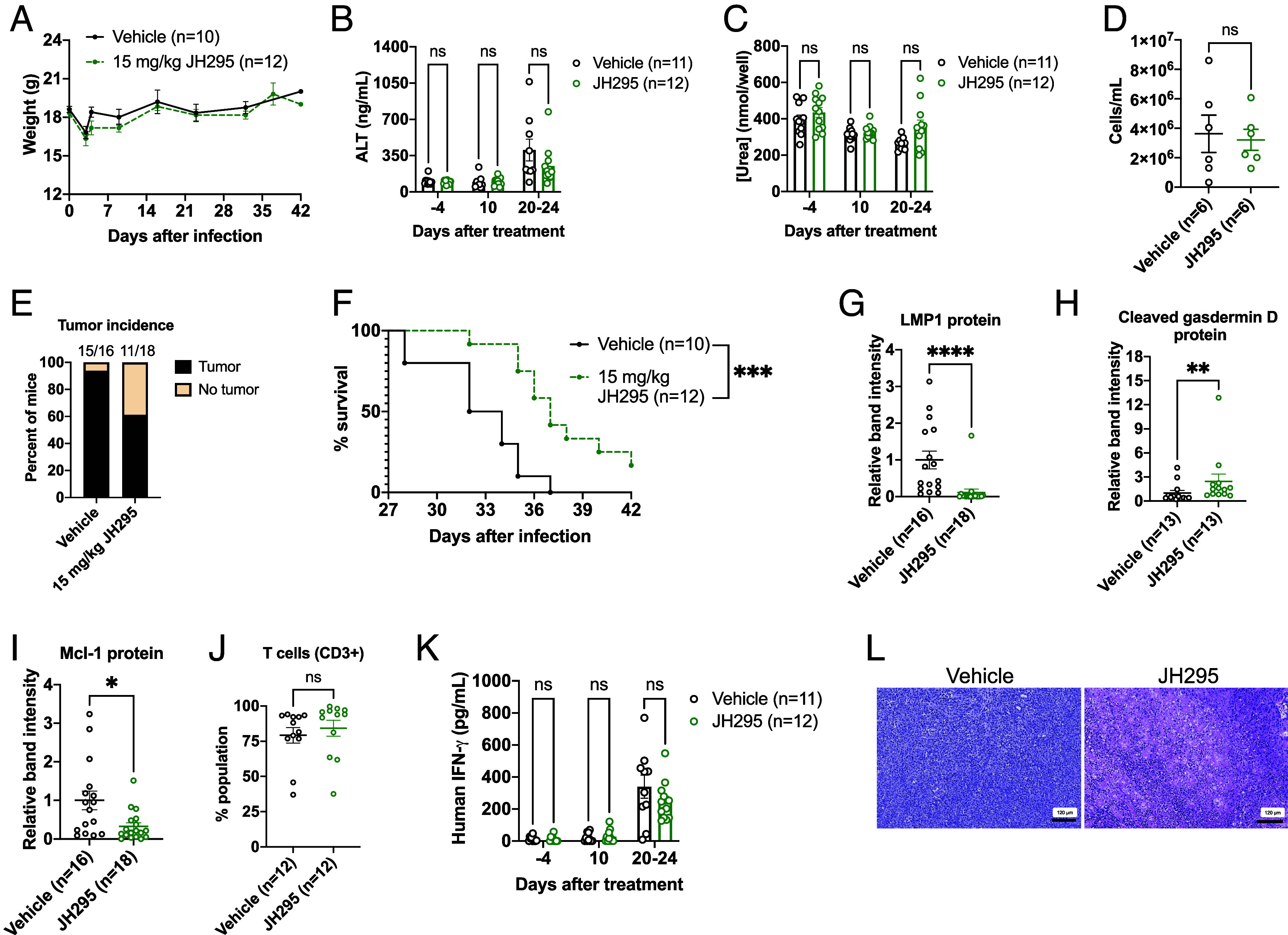
NEK2 inhibition decreases tumor incidence and tumor burden, prolongs survival, and suppresses LMP1 expression in a cord blood-humanized mouse model of EBV-driven lymphomagenesis. (*A*) Weights of mice treated with DMSO or 15 mg/kg JH295. N = 2 biological replicates. (*B*) Serum ALT quantification. N = 2 biological replicates. Data analyzed using mixed-effects analysis with Bonferroni’s multiple comparisons test and graphed mean ± SEM. (*C*) Serum urea quantification. N = 2 biological replicates. Data analyzed using mixed-effects analysis with Bonferroni’s multiple comparisons test and graphed mean ± SEM. (*D*) Splenocyte counts from DMSO- and JH295-treated mice. Representative of N = 2 biological replicates. Data analyzed using the *t* test and graphed mean ± SEM. (*E*) Tumor incidence at necropsy. N = 3 biological replicates. (*F*) Survival curves of mice treated with DMSO or 15 mg/kg JH295. N = 3 biological replicates. Data analyzed using log-rank (Mantel-Cox) test. ***P = 0.0007. (*G*–*I*) Quantification of LMP1 (*G*), cleaved gasdermin D (*H*), and Mcl-1 (*I*) protein from splenic lysates from DMSO- and JH295-treated mice. Immunoblots are in *SI Appendix*. N = 3 biological replicates. Data plotted as mean ± SEM and analyzed using the Mann–Whitney test. ****P < 0.0001; **P = 0.0035; *P = 0.0151. (*J*) Percentage of T cells in the spleens of mice. N = 2 biological replicates. Data plotted as mean ± SEM and analyzed using the *t* test. (*K*) Serum human interferon gamma quantification. N = 2 biological replicates. Data analyzed using mixed-effects analysis with Bonferroni’s multiple comparisons test and graphed mean ± SEM. (*L*) Fixed tumor sections stained with hematoxylin and eosin. Images representative of N = 3 mice per group. (Scale bar, 120 μm.)

Although the above data are promising, one limitation of xenograft models is the lack of immune system components. To study NEK2 inhibition in the presence of functional immune cells, we used a cord blood-humanized mouse model of EBV-driven lymphomagenesis, initially described by the Kenney laboratory (36). In this model, human cord blood mononuclear cells (CBMNCs) are infected ex vivo with EBV and then injected intraperitoneally into NSG mice. Within the host, the infected human B cells become transformed by EBV and initiate systemic lymphomagenesis at near 100% incidence, with humane endpoints being reached within 4 to 6 wk. Importantly, functional human T cells also engraft in this model (36). NSG mice were injected with >5 million EBV-infected human CBMNCs. 7 d postinjection, mice were randomized and treated with DMSO or 15 mg/kg JH295 until humane endpoints were reached (survival, protein analyses) or 4 wk post-CBMNC injection (cellular analyses, histology). Similar to *SI Appendix*, Fig. S7*A*, JH295 had no adverse effects on the weights of the mice (Fig. 6*A*). To further assess potential JH295 toxicity, we assessed liver and kidney function prior to treatment initiation and in 2-wk intervals after starting treatment using sera obtained via facial vein bleed. JH295 had no significant effect on liver function (ALT as proxy; Fig. 6*B*) or kidney function (urea as proxy; Fig. 6*C*) over time compared to control mice, suggesting JH295 is not toxic. Furthermore, splenocyte counts revealed no difference in cell numbers in the presence or absence of JH295 (Fig. 6*D*), supporting a lack of toxicity. Interestingly, we found 94% of the vehicle-treated mice had observable tumors at necropsy, while only 61% of the JH295-treated mice had observable tumors (Fig. 6*E*). Considering the aggressiveness of this mouse model, this suggests JH295 reduces EBV-positive lymphoma incidence in vivo. Additionally, JH295 treatment resulted in a significant increase in overall survival (Fig. 6*F*). Mechanistically, we found JH295-treated animals had decreased LMP1 RNA and protein expression (Fig. 6*G* and *SI Appendix*, Fig. S8 *A* and *B*), increased cleaved gasdermin D protein expression (Fig. 6*H* and *SI Appendix*, Fig. S8*C*), and decreased Mcl-1 protein expression (Fig. 6*I* and *SI Appendix*, Fig. S8*D*) compared to DMSO-treated mice, recapitulating our cell culture findings. Expression of other EBV proteins, including EBNA1, LMP2A, and EBNA2, were not altered by JH295 in vivo (*SI Appendix*, Fig. S8 *E*–*G*). Additionally, we observed no significant effect of JH295 on the proportion of T cells (Fig. 6*J*), B cells (*SI Appendix*, Fig. S8*H*), or live and total human CD45+ cells (*SI Appendix*, Fig. S8*I*) present in the spleens of the animals, suggesting JH295 does not target untransformed immune cells in vivo. Furthermore, our findings indicate JH295 does not affect immune system functional capacity, as quantification of systemic interferon gamma over time was not significantly altered (Fig. 6*K*), nor was RNA or protein expression of interferon gamma at endpoint (*SI Appendix*, Fig. S8 *J* and *K*). Finally, hematoxylin and eosin staining of fixed tumor tissue revealed JH295 resulted in more dead and dying cells within the tumor compared to DMSO (Fig. 6*L* and *SI Appendix*, Fig. S8*L*), suggesting inhibition of NEK2 results in EBV-positive NHL cell death in vivo. Altogether, these data suggest JH295 reduces both the incidence and burden of EBV-positive NHL in animals with no observable toxicity, and significantly improves host survival.

### JH295 Sensitizes EBV-Positive NHL to Doxorubicin.

Considering the ongoing challenge of drug resistance in the clinical management of EBV-positive malignancies, we tested if JH295 rendered cells more susceptible to other chemotherapies. We treated cells with JH295, doxorubicin (used to treat NHL), or JH295 plus doxorubicin and measured cell viability. We found combination therapy of JH295 plus doxorubicin resulted in more cell death than either drug when used alone (*SI Appendix*, Fig. S9*A*). When cells were treated with a range of doxorubicin concentrations in the presence and absence of JH295, we found the addition of JH295 resulted in a curve shift, defining JH295-mediated doxorubicin sensitization (*SI Appendix*, Fig. S9*B*). Overall, these data suggest JH295 can sensitize EBV-positive NHL to drugs already used clinically to treat EBV-associated malignancies.

## Discussion

This report describes the importance of NEK2 in EBV-positive NHL pathogenesis. We show primary EBV infection increases NEK2 expression in human B cells and that the EBV latency proteins EBNA1, EBNA2, and LMP1 independently drive NEK2 expression. We demonstrate both genetic depletion and pharmacologic inhibition of NEK2 result in significant EBV-positive NHL cell death. Additionally, we show EBV-positive NHL is significantly more susceptible to NEK2 inhibition compared to matched EBV-negative NHL. We identify Bcl-2, Mcl-1, Bcl-xL, beta-catenin, and c-myc as cell survival proteins that are modulated by NEK2 signaling to promote EBV-positive NHL growth. We also demonstrate expression of LMP1 is maintained by NEK2 signaling. We show treatment with the NEK2-specific inhibitor, JH295, reduces the expression and function of the drug resistance proteins, MDR1 and MRP1, in EBV-positive lymphomas. Additionally, we identify MRP1 as the ABC transporter protein most active in EBV-positive NHL. Furthermore, we show treatment of mice with JH295 reduces tumor burden of various EBV-positive NHL subtypes and, in a cord blood-humanized mouse model of EBV-driven lymphomagenesis, significantly prolongs survival and reduces tumor incidence without detrimental effects to body weight, organ function, and immune cell function. Finally, we find JH295 sensitizes EBV-positive NHL to doxorubicin and, when both drugs are combined, result in greater cell death than either drug alone. Taken together, our data suggest targeting NEK2 could be a viable therapeutic strategy in EBV-positive NHL.

Like all herpesviruses, EBV establishes latency in host cells during which minimal viral genes are expressed, allowing the virus to evade the immune system and persist throughout the lifetime of the host. EBV has three distinct latency phases classified by viral protein expression, where latency I expresses the fewest proteins and latency III expresses the most. In this study, we chose cell lines that represented all three EBV latency stages: BL (latency stage I); NKTL (latency stage II); and PTLD and LCL (latency stage III). Our data show NEK2 inhibition is effective across all latency stages of EBV. One differentially expressed gene during EBV latency (latency II and III) is LMP1, a major EBV oncoprotein necessary for B cell transformation (37). Interestingly, we observed a decrease in LMP1 expression in all latency stage II and III cell lines following NEK2 inhibition, while expression of EBNA1, an EBV latency protein present across all latency stages, was unchanged. As LMP1 prevents multiple forms of cell death in infected cells (38, 39), it is tempting to speculate JH295 mediates lymphoma cell death by suppressing LMP1 signaling. However, we also observed robust cell death in the latency stage I BL cell lines, which do not express LMP1. Therefore, JH295-mediated cell death cannot be solely attributed to loss of LMP1 expression. We also observed a decrease in Mcl-1 and Bcl-2 expression following JH295 treatment. While LMP1 does induce expression of these proteins to promote infected cell survival (40), their expression is also controlled by beta-catenin signaling (41, 42). Beta-catenin is directly phosphorylated by NEK2 and is a main NEK2 target (43). Notably, the phenotypes we observe in EBV-positive NHL following NEK2 inhibition (including decreased survival, dysregulated cell cycle, and decreased drug resistance) are consistent with decreased expression and signaling capacity of beta-catenin in cancer (44). Additionally, we found EBNA1 upregulates NEK2 protein in EBV-negative lymphoma cells (*SI Appendix*, Fig. S1 *B* and *C*). Together, these data demonstrate how the relationship between NEK2 and beta-catenin signaling can account for the phenotypes observed in BL with JH295 in the absence of LMP1.

Our data suggest JH295 mediates inflammatory cell death in EBV-positive NHL. We observed an increase in LDH release from cells 4 h posttreatment, suggesting JH295 initiates plasma membrane deterioration early in the treatment course. Notably, LDH release was not observed in JSC1, a cell line that undergoes apoptosis with JH295 (15). During the inflammatory cell death mechanism of pyroptosis, gasdermin D is cleaved by caspase 1 and forms pores in the plasma membrane, thus compromising its integrity and allowing for LDH release (45). Levels of cleaved gasdermin D were increased in EBV-positive NHL following NEK2 inhibition; however, we were unable to detect cleaved caspase 1 or cleaved IL-1 protein following JH295 treatment. Therefore, we could not confirm pyroptosis and instead refer to the observed cell death as an inflammatory, non-apoptotic mechanism. Interestingly, we observed an EBV-specific increase in ROS following NEK2 inhibition, which can be associated with inflammatory cell death in cancer (46). A previous study in gastric cancer reported NEK2 inhibition resulted in increased ROS production and inflammatory cell death (23). Additionally, JH295 resulted in a significant decrease in lactate, a molecule that can facilitate adaptation to oxidative stress in cancer (47). Finally, we used the drug, JC2-11, to confirm inflammatory cell death in EBV-positive NHL following NEK2 inhibition. JC2-11 is a synthetic compound analogous to chalcone, found in anti-inflammatory flavonoids inherent to fruits, vegetables, and teas (48). JC2-11 decreases inflammation, in part by inhibiting ROS production, gasdermin D cleavage, and LDH release (49), all observed in EBV-positive NHL following JH295 treatment. When we treated the cells with JH295 in combination with JC2-11, we were able to partially rescue JH295-mediated cell death in EBV-positive NHL but not EBV-negative NHL. Taken together, these data suggest inflammatory cell death in EBV-positive NHL following NEK2 inhibition. We also found expression of the anti-apoptotic proteins, Bcl-2, Mcl-1, and Bcl-xL, were decreased with JH295, even in the absence of apoptosis. Bcl-2, Mcl-1, and Bcl-xL can be modulated by beta-catenin, either directly or through downstream signaling (50–52), and their canonical role is to prevent apoptosis. Although protein levels of Bcl-2 family members are not always equivalent to their activity, we found inhibition of these proteins resulted in cell death, suggesting Bcl-2, Mcl-1, and Bcl-xL all support EBV-positive NHL viability outside of their normal anti-apoptotic role. Previous studies have described non-anti-apoptotic roles of Bcl-2, Mcl-1, and Bcl-xL in cancer, including promoting tumor growth and decreasing chemotherapy efficiency (53, 54). Furthermore, both Mcl-1 and Bcl-2 are involved in cell cycle progression and regulation of cellular metabolism (55–57). Taken together, these data support Mcl-1 and Bcl-2 as having alternative pro-survival roles in EBV-positive NHL aside from counteracting apoptosis.

We demonstrate JH295 induces death of EBV-positive NHL but not PBMCs or primary B cells. When B cells were stimulated to rapidly proliferate and treated with JH295, cell death was 100 to 1,000-fold lower than in EBV-positive NHL. Although all dividing cells express NEK2 and likely utilize NEK2 for cell cycle progression and mitosis, we postulate loss of NEK2 signaling in normal cells, but not lymphoma cells, can be compensated for by other mitotic kinases. Furthermore, we detected no toxicity of JH295 in vivo, as liver function, kidney function, body weight, splenocyte count, T cell number, human CD45 cell engraftment and survival, and interferon gamma expression in treated animals did not differ from control animals, supporting feasibility of this therapeutic approach.

We found NEK2 inhibition decreased expression and activity of the drug resistance proteins, MDR1 and MRP1. Interestingly, both MDR1 and MRP1 are downstream targets of NEK2 (12). Accordingly, NEK2 inhibition has been shown to decrease cisplatin and radio-resistance in cervical cancer (58, 59). Drug resistance represents one of the most challenging obstacles in cancer therapy, especially for NKTL. NKTLs are widely known to be highly resistant to multiple treatment modalities, particularly anthracyclines, which include doxorubicin (60–62). Here, we show MRP1 is the ABC transporter most active in EBV-positive NHL. MDR1 was also active in NKTL, but to a lesser extent than MRP1. We found a vast majority of substrate was pumped out of cells through MRP1 in the presence and absence of JH295. While the ABC transporter inhibitors used can have potential off-target effects, the inhibitors were only in contact with the cells for a short period of time and, once removed, cells were immediately processed for flow cytometry. Additionally, we observed a significant decrease in overall drug efflux activity in EBV-positive NHL following NEK2 inhibition, resulting in more substrate being retained within the cells. These data were especially striking for the SNK6 and SNT16 cell lines, as NEK2 inhibition blocked MRP1-mediated efflux almost completely. Notably, SNK6 and SNT16 cells are resistant to chemotherapy, including etoposide and doxorubicin (61, 62). Together, these data suggest not only can JH295 induce EBV-positive NHL death, but it can also lower drug resistance, leading us to hypothesize JH295 could sensitize lymphoma cells to other drugs currently used clinically. Indeed, we found JH295 treatment sensitized the cells to doxorubicin, a component of the widely used NHL therapy, R-CHOP, highlighting the potential use of JH295 in combinatorial cancer therapy.

Here, we used a cord blood-humanized mouse model of EBV-driven lymphomagenesis to test the effects of NEK2 inhibition in vivo, as EBV-mediated B cell transformation occurs inside the host and drives systemic lymphoma development, similar to how these lymphomas develop in humans. Notably, these tumors are not palpable, as they are often embedded within the mesentery and invade visceral organs. As such, this lymphoma mouse model is highly aggressive and results in near 100% tumor incidence. We found JH295 decreased tumor incidence in mice (control 94%; JH295 61%), reduced tumor burden, and prolonged survival. We observed similar mechanistic phenotypes in vivo as in cell culture, including decreased Mcl-1 and LMP1 expression and increased cleaved gasdermin D expression following JH295 treatment. LMP1 was the only EBV latency protein downregulated by NEK2 inhibition in vivo, as we found no differences in EBNA1, LMP2A, or EBNA2 expression in vehicle versus JH295-treated animals. We also observed JH295-mediated cell death within the tumor tissue, suggesting intraperitoneal administration of JH295 successfully reaches these systemic lymphomas. Promisingly, liver and kidney function remained unaffected by JH295 in the mice, as did T cell number and interferon gamma production. It is still unclear which functional immune cell subsets engraft in this mouse model aside from T cells, but T cells are one of the main producers of interferon gamma in response to antigen (63). Although we were unable to definitively determine if the interferon gamma response was virus-specific or tumor-specific, our data indicate an antitumor immune response, as the kinetics of interferon gamma production matched those of tumor development and not viral infection (Fig. 6*K*). Therefore, JH295 does not appear to negatively target immune cells responding to tumor antigen, even though these cells would likely be proliferating.

In conclusion, we present NEK2 as a therapeutic target for EBV-positive NHL. Genetic and pharmacologic targeting of NEK2 resulted in EBV-positive lymphoma cell death, and EBV-positive NHL were significantly more reliant on NEK2 signaling for survival than matched EBV-negative NHL. NEK2 inhibition also reduced tumor burden and prolonged survival in a humanized mouse model of EBV-driven lymphomagenesis. Our data also suggest NEK2 inhibition could be a powerful tool to help mitigate the enduring challenge of drug resistance in cancer treatment.

## Materials and Methods

### Cells.

Cells were grown in RPMI 1640 medium (Corning) supplemented with 10% FBS, 1% penicillin-streptomycin, and 1% L-glutamine. Akata-BX1 media contained 1% Geneticin (Gibco); NKTL media contained 800 U/mL human IL-2.

### NEK2 RNA Expression in PBMCs.

Data are from the Gene Expression Omnibus (GEO) repository (NCBI; GSE235941). Accession numbers can be found in *SI Appendix*.

### NEK2 Transcripts in Human Tumors.

GEO repository data were analyzed using the R2 Genomics Analysis and Visualization platform. Accession numbers can be found in *SI Appendix*.

### EBV Plasmid Transfections.

BJAB cells were transfected with EBV open reading frame plasmids (VectorBuilder or Addgene) using the Amaxa Cell Line Nucleofector Kit (Lonza) according to the manufacturer’s instructions. Cells were incubated for 2.5 to 3 d and then harvested for immunoblotting.

### NEK2 Knockdown.

Cells were spinfected with lentivirus expressing Sigma Mission® NEK2 shRNAs or control plasmid. The ViraPower™ Lentiviral Packaging Mix (ThermoFisher) was used according to the manufacturer’s instructions. Cells were harvested 72 h later for use in assays.

### Western Blotting.

Whole cell samples were lysed in 0.1% NP40 lysis buffer and resolved via SDS-PAGE. In some instances, blots were probed for multiple proteins and the loading control was the same for these proteins as indicated in the corresponding figure legends. Antibodies used are in *SI Appendix*.

### Primary B Cell Assays.

B cells were isolated from PBMCs using the STEMCELL EasySep system. For stimulation, cells were treated with 50 ng/mL recombinant human CD40L (R&D Systems) and 20 ng/mL recombinant human IL4 (R&D Systems) for 24 h, then treated with JH295 for an additional 48 h.

### Multidrug Resistance Assays.

MFI was calculated on the single-cell PI-negative population. To calculate the multidrug resistance activity factor (MAF), the following equation was used: 100 − {100 * [(MFI_sample_ − MFI_DMSO control_)/MFI_sample_]}.

### SNK6 Xenograft Model.

All animal experiments were approved by the Institutional Animal Care and Use Committee at the University of North Carolina at Chapel Hill. NSG mice were subcutaneously injected with 1 million SNK6 cells. Mice were randomized and treated with DMSO (vehicle control) or 15 mg/kg JH295 intraperitoneally for 23 d. Tumor volume was calculated using the following formula: π × length × width × height/6.

### Cord Blood-Humanized Mouse Model of EBV-Driven Lymphomagenesis.

NSG mice were injected with >5 million EBV-infected CBMNCs. 7 d later, mice were randomized and treated with DMSO or 15 mg/kg JH295 intraperitoneally until endpoints were reached. Each experiment used a unique cord blood donor.

### ALT, Urea, and Interferon Gamma Assays.

Kits (Abcam) were used following the manufacturer’s instructions. Serum was diluted 1:340 for the ALT assay; 1:150 for the urea assay; and used neat or diluted as little as possible for the interferon assay.

### Histology.

Paraffin-embedded formalin-fixed slides of EBV-positive tumors were generated by the Pathology Services Core at the University of North Carolina at Chapel Hill and stained with hematoxylin and eosin. Subsequent analyses were verified by a pathologist.

### Statistics.

Analyses were performed using GraphPad Prism 9. Unless otherwise stated, experiments were performed three independent times on different days.

## Supplementary Material

Appendix 01 (PDF)

## Data Availability

All study data are included in the article and/or *SI Appendix*.
