# Supplementary material for: NEK2 drives pathogenesis, drug resistance, and LMP1 expression in EBV-positive non-Hodgkin lymphoma
Source: Proc Natl Acad Sci U S A. 2026 May 14;123(20):e2535550123. doi: 10.1073/pnas.2535550123 (PMC13187809; doi:10.1073/pnas.2535550123)
Supplement: Supplementary file 1 — Appendix 01 (PDF) [file pnas.2535550123.sapp.pdf]

PNAS

Supporting Information for

NEK2 drives pathogenesis, drug resistance, and LMP1 expression in EBV-positive non-Hodgkin lymphoma

Maria C. White[1,2], Philip T. Lange[1,2], Jessica Stewart[1,2], and Blossom Damania[1,2,*]

Blossom Damania
Email: damania@med.unc.edu

This PDF file includes:

Supporting text
Figures S1 to S9
Table S1 to S3
SI References

Supporting Information Text

Materials and Methods

Cell lines and reagents PBMCs and CBMNCs were purchased from STEMCELL. Cells were used for experiments when in log phase and were not kept in culture for more than 10 weeks. Primary B cells were isolated from commercially obtained peripheral blood mononuclear cells (PBMCs) using the STEMCELL EasySep system (17954), and all biological replicates used unique cell donors. Cord blood mononuclear cells were used immediately after thawing. Each mouse experiment used a unique cord blood donor.

JH295 was purchased from Tocris (4322); NBI-961 was purchased from MedChemExpress (HY-156437); NCL 00017509 was purchased from Tocris (5150); JC2-11 was purchased from MedChemExpress (HY-152670); the Mcl-1/Bcl-2-IN-2 dual inhibitor was purchased from MedChemExpress (HY-129700); MSAB was purchased from MedChemExpress (HY-120697); NCB-0846 was purchased from MedChemExpress (HYH-100830); A-1155463 was purchased from Selleckchem (S7800); $DiOC_2(3)$ (3,3'-Diethyloxacarbocyanine Iodide) was purchased from ThermoFisher Scientific (Invitrogen, D14730); verapamil hydrochloride was purchased from MedChemExpress (HY-A0064); MK-571 sodium was purchased from MedChemExpress (HY-19989A); novobiocin was purchased from MedChemExpress (HY-B0425A); doxorubicin was a kind gift from Dirk Dittmer.

For mouse experiments, sterile 100% DMSO (20 µL injection volume) was used as the vehicle control. JH295 was used at a concentration of 15 mg/kg; assuming animal weight of ~0.02 kg, each mouse received 0.3 mg JH295 in 20 uL injection volume. Drugs were prepared weekly for injection.

EBV infection of primary B cells Primary B cells were infected with 11,000 green Raji units of EBV. After 48h, cells were imaged using a Leica DMi8 inverted microscope and harvested for protein isolation.

NEK2 RNA expression in EBV-infected PBMCs

Data are from the Gene Expression Omnibus (GEO) repository (NCBI; GSE235941). GEO2R was used to compare groups to identify differentially expressed genes across experimental conditions. Accession numbers: GSM7512208 (control); GSM7512209 (two days post-infection replicate 1); GSM7512210 (two days post-infection replicate 2); GSM7512211 (four days post-infection replicate 1); GSM7512212 (four days post-infection replicate 2).

NEK2 transcripts in human tumors Data were obtained using the Affymetrix Human Genome U133 Plus 2.0 Array platform and normalized with the MAS5.0 algorithm to allow for comparisons between datasets. Data were analyzed using the R2 Genomics Analysis and Visualization platform. GEO accession GSE12366 (Jima); GSE26673 (Piccaluga); GSE168422 (Chan); GSE38885 (Bittoun); GSE20874 (Dykema).

NEK2 knockdown Sigma MISSION® shRNAs were used to generate lentivirus particles. pLKO.1-puromycin control (Sigma, SHC002) and the following NEK2 shRNAs in the pLKO.1 backbone (Sigma, SHCLNG-NM_002497) were used: TRCN0000000948 (NKTL) and TRCN0000195573 (BL, PTLD). Cells were spinfected with 0.5 mL lentivirus/well in duplicate wells in the presence of polybrene (10 µg/mL; Sigma, TR-1003). Media was replaced with selection media (1 µg/mL puromycin [Corning, 61385RA]) the next day. Cells were kept under puromycin selection for the duration of the assay.

For GM12878 cells, which are not amenable to lentivirus transduction, ON-TARGETplus Human NEK2 SMARTpool siRNA (Dharmacon, 4751) was used to deplete NEK2 protein (J-004090-18: GGAUCUGGCUAGUGUAAUU, J-004090-19: GCAGACAGAUCCUGGGCAU, J-004090-20: GGCAAUACUUAGAUGAAGA, and J-004090-21: GCUAGAAUAUUAAACCAUG). The ON-TARGETplus control pool siRNA (Dharmacon, D-001810-10-20) was used as the nontemplate control (NTC). The Amaxa Cell Line Nucleofector Kit (Lonza, VCA-1003) was used according to the manufacturer's instructions. Cells were harvested 48h later for use in assays.

Cell viability assays CellTiter-Glo (CTG) Cell Viability assays (Promega, G7573) were performed according to the manufacturer's instructions using 25 microliters of prepared reagent. Luminescence

was recorded using a CLARIOstar Plus Plate Reader (BMG Labtech). One plate was seeded per time point assayed. A 0h plate was included for luminescence data normalization when appropriate.

GI50 determination Cells were treated with a range of the indicated drug or DMSO control for 48h and then cell counts were performed via trypan blue staining. The GI50 for each drug for each cell line was then calculated using the following formula, where T_0 = seeded cell count; C = cell count of control sample at experiment end; and T_X = cell count of experimental sample at experiment end: $100*((T_X-T_0)/(C-T_0))$.

Western blotting Protein was quantified using Bradford Assay (Bio-Rad Protein Assay Dye, 5000006). Prepped protein lysates were boiled for 5-10 minutes at 95 ºC. Antibodies were purchased from either Cell Signaling Technology (CST) or Santa Cruz. Primary antibodies were diluted 1:1000 except for GAPDH, which was diluted 1:2500. NEK2 (Santa Cruz, 55601); GAPDH (Santa Cruz, 47724); Vinculin (Santa Cruz, 25336); Gasdermin D (CST, 97558); Bcl-2 (CST, 4223); Mcl-1 (CST, 5453); Bcl-xL (CST, 2764S); MDR1 (CST, 13342S); MRP1 (CST, 72202S); BCRP (CST, 42078S); total beta-catenin (CST, 8480S); phosphorylated beta-catenin S33/37/T41 (CST, 9561S); phosphorylated beta-catenin S675 (CST, 4176S); and c-myc (CST, 13987S). The EBV antibodies were purchased from Abcam or Santa Cruz. LMP1 (Abcam, ab78113); EBNA2 (Abcam, ab90543); EBNA1 (Santa Cruz, sc81581); and LMP2A (Santa Cruz, sc101314). Secondary antibodies were diluted 1:2000. Anti-rabbit IgG-HRP (CST, 7074S); anti-rat IgG-HRP (CST, 7077S); and anti-mouse IgG-HRP (CST, 7076S).

For the Ponceau S protein staining, Ponceau S staining solution (Sigma, P7170) was applied to the full nitrocellulose membrane for several minutes, then rinsed twice with 1XTBST prior to image collection.

Primary cell assays The purity of isolated B cells was verified by flow cytometry (MACSQuant VYB, Miltenyi Biotech). Alexa Fluor 594 Anti-Human CD3 antibody (BioLegend, 300446) and Pacific Blue Anti-Human CD20 antibody (BioLegend, 302319) were used to verify purity. B cell populations of >95% purity were used.

Propidium iodide (PI) flow viability assays Cells were treated with and without JH295 for 48h and then 1.5 million cells per sample were resuspended in cell staining buffer (BioLegend, 420201)

containing PI (BioLegend, 421301) and run on a MACSQuant® VYB flow cytometer (Miltenyi Biotec) to quantify PI-positive cells. Events were gated on the single cell population.

Cell cycle assays Cell cycle assays were performed as described previously (1).

LDH and ROS (H_2O_2) assays The LDH-cytotoxicity assay kit (Abcam, ab197004) and the ROS-Glo H_2O_2 assay (Promega, G8820) were used according to the manufacturer's instructions.

Lactate assays The Lactate-Glo assay (Promega, J5021) was used according to the manufacturer's instructions. After briefly shaking the plate, 7 μL was taken from each well (containing both cells and media) and added to 195 μL PBS. After mixing, 12 μL of each diluted sample or standard was transferred to clean wells and combined with 12 μL assay solution. The dilution factor was corrected for during lactate concentration calculation.

Multidrug resistance assays The protocol for the multidrug resistance assay was based on an existing protocol (Sigma, ECM910) as described in (1) using independently acquired $DiOC_2(3)$. Cells were treated with JH295 for 48h, then loaded with $DiOC_2(3)$ on ice for 15-20 minutes. Cells were then moved to 37° C, the temperature at which ABC transporters are active, and substrate efflux ocurred over 1.5-2h. A verified 37 ºC water bath was used for the efflux step. A control was included in the assay which maintained a maximum amount of dye within the cells by continuously keeping the cells on ice. PI was used to gate out dead cells.

eFLUXX-ID® Green assays The eFLUXX-ID® Green Multidrug Resistance assay kit (Enzo, ENZ-51029-K100) was used according to the manufacturer's instructions. Reagents were reconstituted using anhydrous DMSO (Biotium, 90082). Cells were seeded in complete phenol red-free RPMI medium (Gibco, 11835-030) containing appropriate supplements. Dye loading (10 minutes) and dye efflux (75 minutes) were performed in a verified 37 ºC water bath protected from light. The green dye is fluorescent only after it enters the cells and is cleaved by cellular esterases that are active at 37 ºC. A sample loaded with dye but constantly kept on ice thereafter served as the negative control.

PBMC immunoblotting Whole cell samples from two unique donors were lysed in 0.1% NP40 lysis buffer and cleared by centrifugation. Protein was quantified using Bradford Assay (Bio-Rad Protein

Assay Dye, 5000006). Prepped protein lysates were boiled for 5 minutes at 95 ºC. Proteins were resolved via SDS-PAGE. Primary antibodies were diluted as follows: MDR1, beta-catenin, Bcl-2, and Mcl-1 1:500; Bcl-xL 1:700; and GAPDH 1:1650.

SNK6 xenograft mouse model The SNK6 xenograft experiments were performed by the Preclinical Research Unit (PRU) at the University of North Carolina at Chapel Hill. NSG (strain name NOD.Cg-Prkdc^{scid}Il2rg^{tm1Wjl}/SzJ) mice weighing approximately 20 g were purchased from The Jackson Laboratory by the PRU. Mice were housed under pathogen-free conditions and had free access to food and water. Female NSG mice, 6-8 weeks of age, were injected subcutaneously in the right flank with 1 million SNK6 cells in a 1:1 ratio of PBS:Matrigel (Corning). Following tumor engraftment, mice were randomized according to tumor volume and treated 3x/week (M, W, F) with DMSO (vehicle control) or 15 mg/kg JH295 intraperitoneally for 23 days. Animal weights were taken 3x/week. Tumors were measured 3x/week using calipers.

Cord blood-humanized mouse model of EBV-driven lymphomagenesis Commercially available human cord blood mononuclear cells (CBMNCs) were thawed and treated with DNase (~100 units/mL) prior to infection to prevent clumping. CBMNCs were then infected with 2,500 green Raji units of the Akata-BX1 EBV strain per mouse for one hour at 37 ºC with intermittent agitation. Cells were centrifuged to remove DNase and unadsorbed virus and resuspended in addititve-free RPMI for injection. Female NSG mice, 4-6 weeks of age, were injected intraperitoneally in the lower right quadrant with 200 uL cell solution containing >5 million CBMNCs. Mice were randomized into treatment groups based on weight and treated 3x/week (M, W, F). As tumors in this model are not palpable, humane endpoint was defined by body condition score of 2 or below, jaundice development, impaired mobility, or moderate-extreme lethargy. Animals were weighed weekly, and blood collection prior to necropsy for seralogical analyses (no more than 1% of body weight per sampling day) was obtained via facial vein bleed using a lancet. Facial vein bleeds were spaced out by two weeks and were not performed on treatment days.

ALT, urea, and interferon gamma assays Serum was used to quantify ALT as a proxy for liver function using the Mouse ALT ELISA kit (Abcam); urea as a proxy for kidney function using the Urea Assay kit

(Abcam); and interferon gamma as a proxy for immune system function using the Human Interferon Gamma High Sensitivity ELISA kit (Abcam) following the manufacturer's instructions.

Protein isolation from mouse spleens Half of each mouse spleen was homogenized with 1-mm zirconia beads in 1 mL water using a MagNA Lyser instument (Roche Diagnostics). Homogenate was then diluted 1:1 in 2X radioimmunoprecipitation assay (RIPA) lysis buffer and processed.

Reverse transcription quantitative polymerase chain reaction (RT-qPCR) Half of each mouse spleen was homogenized with 1-mm zirconia beads in 1 mL TRIzol using a MagNA Lyser (Roche Diagnostics). RNA was then isolated from the homogenate following the manufacturer's instructions. 2 μg RNA was DNase treated, split in half, and reverse transcribed using the SensiFAST cDNA synthesis kit (Meridian Bioscience, BIO-65054) either with or without reverse transcriptase. RT+ and RT- cDNA were diluted 1:4 in water and qPCR was performed using SensiFAST SYBR Lo-Rox (Bioline, BIO-94020) and a QuantStudio 6 Flex Real-Time PCR system (Applied Biosciences). Gene expression was normalized to GAPDH and fold changes were calculated using the $2^{-\Delta\Delta Ct}$ method. LMP1 F: CAGTCAGGCAAGCCTATGA; LMP1 R: CTGGTTCCGGTGGAGATGA; IFN-γ F: GCATGTCAGACAGAACTTGAATG; IFN-γ R: GAAGCACCAGGCATGAAATC; EBNA1 F: TACAGGACCTGGAAATGGCC; EBNA1 R: TCTTTGAGGTCCACTGCCG; BALF2 F: GGGCTGTGGCGAGTACCAC; BALF2 R: CGCTGGTCCTGTGTGTCTTG; LMP2 F: AGCTGTAACTGTGGTTTCCATGAC; LMP2 R: GCCCCCTGGCGAAGAG.

In vivo *flow cytometry* A single cell suspension of splenocytes was generated from each mouse spleen. Cells were treated with Human TruStain FcX (BioLegend, 422302) and subsequently labeled for flow cytometry using the following antibodies: PE anti-human CD3 (BioLegend, 300308); PE/Dazzle594 anti-human CD19 (BioLegend, 363032); and FITC anti-human CD45 (BioLegend, 368508). The viability dye eFluor 780 (eBioscience, 65-0865-14) was included to identify live cells. Cells were fixed and run on a Becton Dickinson LSRFortessa in the UNC Flow Cytometry Core Facility. Events were gated on the single cell and live cell populations.

Statistical analyses T test was used for two variables and ANOVA was used for three or more variables. p values <0.05 were considered significant. Post-hoc analyses were included where appropriate and are stated in the figure legends. For the animal experiments, sample sizes were determined using G*Power software for 85-95% power to detect a two-fold difference between groups using a 0.05 alpha value and Wilcoxon-Mann-Whitney test. Western data were processed using Image Lab, and flow cytometry data were processed using FlowJo v10.8.0.

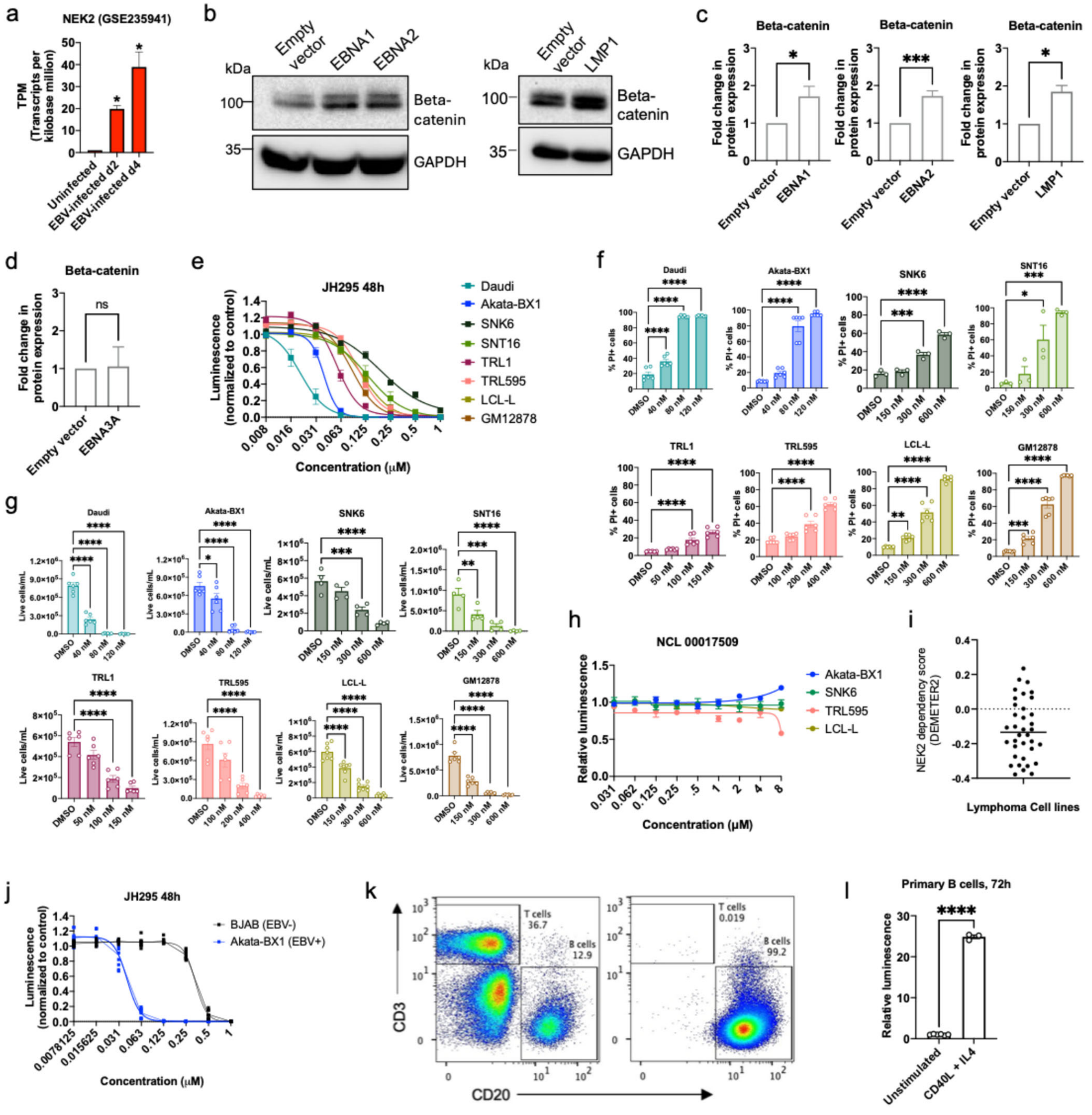

Fig. S1. **EBV latency proteins upregulate beta-catenin expression, and NEK2 signaling supports EBV-positive NHL growth.** (**a**) NEK2 transcripts in uninfected and EBV-infected PBMCs two and four days post-infection. N=2 biological replicates (infected samples) from the Sequence Read Archive. Data were analyzed using GEO2R and plotted as mean ± SEM. *p=4.05e-27. (**b**) Western blot of beta-catenin expression in BJAB cells transfected with EBNA1, EBNA2, LMP1, or empty vector control plasmids.

GAPDH was used as the loading control. Data represent N≥2 biological replicates. (**c-d**) Quantification of beta-catenin protein expression in EBNA1-, EBNA2-, LMP1-, and EBNA3A- transfected BJAB cells, normalized to the corresponding empty vector control. N=2-3 biological replicates. Data were analyzed using t-test and plotted as mean ± SEM. ***p=0.001; *p<0.02; ns=not significant. (**e**) Cell viability curves of JH295 treatment in all eight EBV-positive NHL cell lines. Data were normalized to the DMSO control for each condition. N=3-4 biological replicates performed in triplicate. Curves were fitted using non-linear regression and are plotted as mean ± SEM. (**f**) PI viability assays of EBV-positive NHL treated either with DMSO or increasing concentrations of JH295 for 48h. N=3 biological replicates. Data were analyzed using one-way ANOVA with Dunnett's multiple comparisons test and plotted as mean ± SEM. ****p<0.0001; ***p<0.001; **p=0.0084; *p=0.0134. (**g**) Cell proliferation assays of EBV-positive NHL treated with either DMSO or increasing concentrations of JH295 for 48h. Live cell counts via trypan blue staining were normalized to the initial cell input values. N=3-4 biological replicates. Data were analyzed using one-way ANOVA with Dunnett's multiple comparisons test and plotted as mean ± SEM. ****p<0.0001; ***p=0.0004 SNK6; ***p=0.0002 SNT16; **p=0.0079; *p=0.0319. (**h**) Cell viability curves of EBV-positive NHL treated with the reversible NEK2 inhibitor, NCL 00017509, for 48h. Data were normalized to the DMSO control for each condition. Curves were fitted using non-linear regression and are plotted as mean ± SEM. (**i**) NEK2 dependency in lymphoma cell lines assessed by RNA interference screening. Data were obtained from the DepMap DEMETER2 dataset (Achilles, DRIVE, and Marcotte RNAi screens). Data are graphed as a dot plot showing individual NEK2 dependency scores across lymphoma cell lines; each point represents a single cell line, and the horizontal line indicates the mean dependency score. Negative gene effect scores indicate increased dependency on NEK2. (**j**) Cell viability curves of JH295 treatment in BJAB and Akata-BX1 cell lines. Data were normalized to the DMSO control for each condition. N=3 biological replicates performed in triplicate. Curves were fitted using non-linear regression and plotted independently. (**k**) Flow plots of primary B cell isolation before (left panel) and after (right panel) B cell purification from donor PBMCs. Plots are representative of N=3 biological replicates, each using a unique cell donor. (**l**) Cell viability assay of primary B cells that were

either unstimulated or stimulated with 50 ng/mL CD40L and 20 ng/mL IL4 for 72h. Data were normalized to the unstimulated control. Data were analyzed by t-test and plotted as mean ± SEM. ****$p<0.0001$.

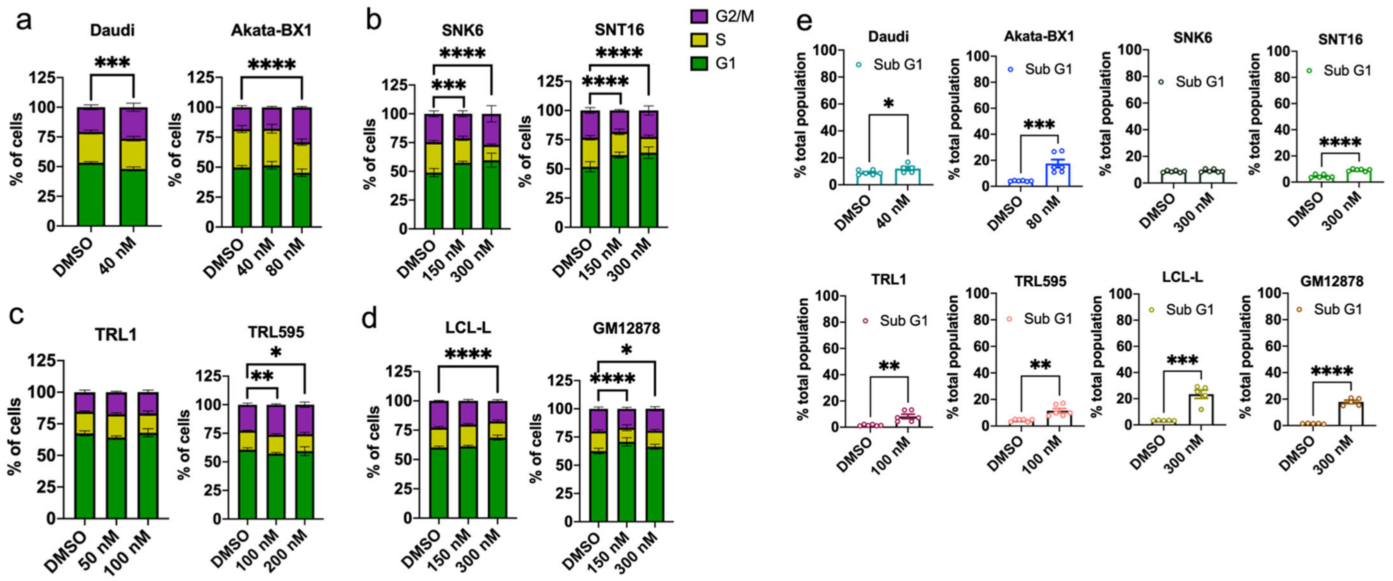

Fig. S2. NEK2 inhibition results in EBV-positive NHL cell cycle arrest. (**a-d**) Cell cycle analysis of EBV-positive lymphoma cells treated with either DMSO or JH295 for 24h. Sub-G1 content was excluded from the analysis. N=3 biological replicates. Data were analyzed using two-way ANOVA with Dunnett's multiple comparisons test and plotted as mean ± SD. (**a**) Analysis of the G2/M population displayed; Daudi dataset analyzed using Sidak's multiple comparisons test. (**b**) Analysis of the G1 population displayed. (**c**) Analysis of the G2/M population displayed. (**d**) Analysis of the G1 population displayed. ****$p<0.0001$; ***$p<0.001$; **$p=0.0021$; *$p<0.015$. (**e**) Analysis of samples in (**a-d**) for sub-G1 content. N=3 biological replicates. Data were analyzed using t-test and plotted as mean ± SEM. ****$p<0.0001$; ***$p=0.0007$ Akata; ***$p=0.0001$ LCL-L; **$p<0.002$; *$p=0.0356$.

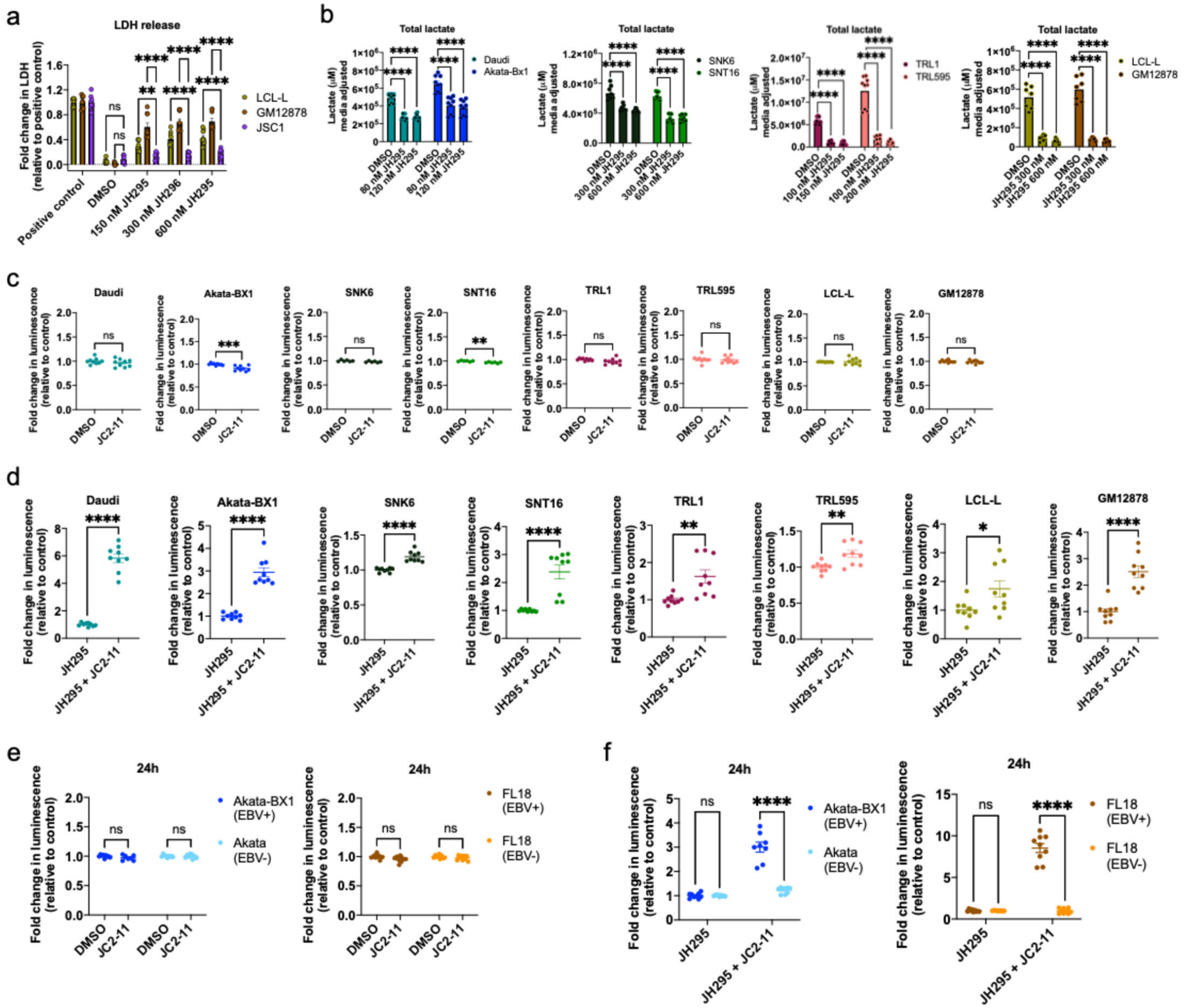

Fig. S3. JH295 treatment induces EBV-positive NHL inflammatory cell death. (a) LDH release assay including same LCL data as in **Fig. 2a** but graphed and analyzed with the JSC1 primary effusion lymphoma cell line data. N=3 biological replicates. Data were analyzed using two-way ANOVA with Tukey's multiple comparisons test and plotted as mean ± SEM. ****p<0.0001; **p=0.004. (**b**) Lactate assays of EBV-positive NHL treated with either DMSO or increasing concentrations of JH295 for 24h. N=3 biological replicates. Data were analyzed using two-way ANOVA with Sidak's multiple comparisons test and plotted as mean ± SEM. ****p<0.0001. (**c and e**) Cell viability assays of EBV-positive NHL (**c**) and coisogenic EBV-positive and EBV-negative cell lines (**e**) treated with either DMSO or 1.5 µM of the

inflammatory corpuscle inhibitor, JC2-11, for 24h. Data were normalized to the 0h luminescence values for each condition and graphed as fold change in luminescence relative to the DMSO control. N=3 biological replicates performed in triplicate. Data were analyzed using t-test (**c**) or two-way ANOVA with Sidak's multiple comparisons test (**e**) and graphed as mean ± SEM. ***p=0.0003 ; **p=0.0013; ns=not significant. (**d and f**) Viability assays of EBV-positive NHL (**d**) and coisogenic EBV-positive and EBV-negative cell lines (**f**) treated with DMSO, JH295, or JH295 + 1.5 μM JC2-11 for 24h. Data were normalized to the 0h luminescence values for each condition and graphed as fold change relative to JH295-only treatment. N=3 biological replicates performed in triplicate. Data were analyzed using t-test (**d**) or two-way ANOVA with Sidak's multiple comparisons test (**f**) and graphed as mean ± SEM. ****p<0.0001; **p=0.0036 TRL1; **p=0.005 TRL595; *p=0.0261. Concentrations of JH295 were used as follows: Daudi=80nM; Akata-BX1=120nM; SNK6 and SNT16=600nM; TRL1=100nM; TRL595=200nM; LCL-L and GM12878=600nM; coisogenic Akata cell lines=125nM; FL18 EBV-positive=62nM; FL18 EBV-negative=500nM.

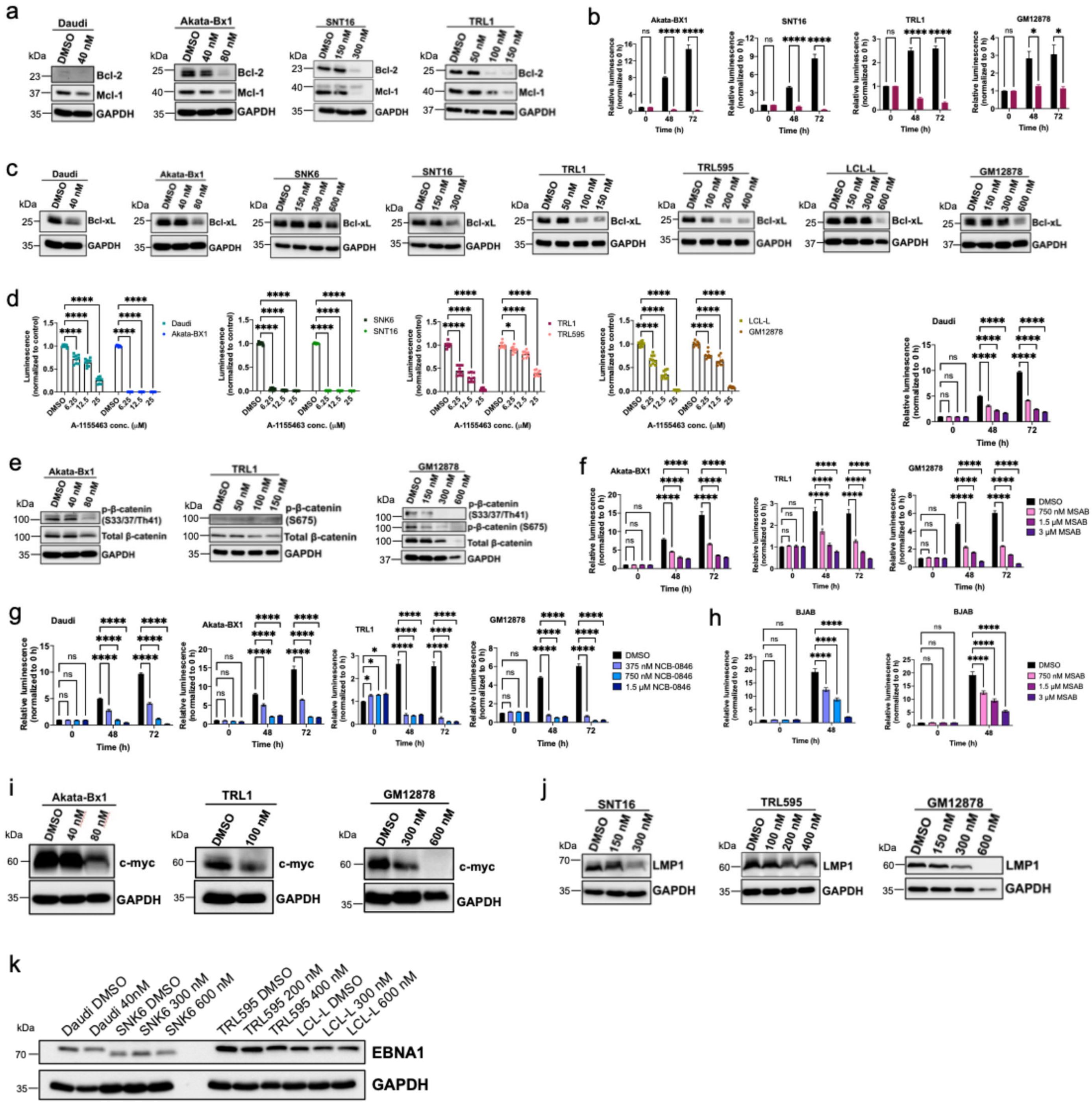

Fig. S4. JH295 treatment of EBV-positive NHL decreases expression of cellular and viral pro-survival proteins. (**a**) Western blot of Mcl-1 and Bcl-2 in EBV-positive NHL treated with either DMSO or increasing concentrations of JH295 for 48h. GAPDH was used as the loading control. Data are representative of N=2-3 biological replicates except for Daudi Bcl-2 (N=1; very low expression). (**b**) Cell viability assays of EBV-positive NHL treated with either DMSO or the Mcl-1/Bcl-2 dual inhibitor over a

72h time course. Data were normalized to the 0h luminescence values for each condition. N=3 biological replicates performed in triplicate. Data were analyzed using two-way ANOVA with Sidak's multiple comparisons test and graphed as mean ± SEM. ****$p<0.0001$; *$p<0.02$. (**c**) Western blot of Bcl-xL in EBV-positive NHL treated with either DMSO or increasing concentrations of JH295 for 48h. GAPDH was used as the loading control. Data are representative of N=2-3 biological replicates. (**d**) Cell viability assays of EBV-positive NHL treated with either DMSO or increasing concentrations of the Bcl-xL inhibitor, A1155463, for 48h. N=3 biological replicates performed in triplicate. Data were analyzed using two-way ANOVA with Dunnett's multiple comparisons test and graphed as mean ± SD. ****$p<0.0001$; *$p=0.014$. (**e**) Western blot of phosphorylated and total beta-catenin in EBV-positive NHL treated with either DMSO or increasing concentrations of JH295 for 48h. GAPDH was used as the loading control. Data are representative of N=2-3 biological replicates. For total beta-catenin, the western blot for phosphorylated beta-catenin was stripped and reprobed with antibody against total beta-catenin. (**f**) Cell viability assays of EBV-positive NHL treated with either DMSO or increasing concentrations of the beta-catenin inhibitor, MSAB, over a 72h time course. Data were normalized to the 0h luminescence values for each condition. N=3 biological replicates performed in triplicate. Data were analyzed using two-way ANOVA with Dunnett's multiple comparisons test and graphed as mean ± SEM. ****$p<0.0001$; ns=not significant. (**g**) Cell viability assays of EBV-positive NHL treated with either DMSO or increasing concentrations of the beta-catenin transcription inhibitor, NCB-0846, over a 72h time course. Data were normalized to the 0h luminescence values for each condition. N=3 biological replicates performed in triplicate. Data were analyzed using two-way ANOVA with Dunnett's multiple comparisons test and graphed as mean ± SEM. ****$p<0.0001$; *$p<0.05$; ns=not significant. (**h**) Cell viability assays of BJAB cells treated with either DMSO or increasing concentrations of the two different beta-catenin inhibitors over time. Data were normalized to the 0h luminescence values for each condition. N=2 biological replicates performed in triplicate. Data were analyzed using two-way ANOVA with Dunnett's multiple comparisons test and graphed as mean ± SEM. ****$p<0.0001$; ns=not significant. (**i**) Western blot of c-myc in EBV-positive NHL. GAPDH was used as the loading control. Data are representative of N=3

biological replicates. (**j**) Western blot of LMP1 in EBV-positive NHL treated with either DMSO or increasing concentrations of JH295 for 48h. GAPDH was used as the loading control. Data are representative of N=2-3 biological replicates. (**k**) Western blot of EBNA1 in EBV-positive NHL treated with either DMSO or increasing concentrations of JH295 for 48h. GAPDH was used as the loading control. Data are representative of N=2 biological replicates. Akata-BX1 loading controls in (**c**) and (**i**) are the same. SNK6, LCL-L, and GM12878 loading controls in (**c**) and **Fig. 2c** are the same. TRL595 loading controls in (**c**), **Fig. 3d**, and **Fig. 4a** are the same. SNT16 loading controls in (**c**) and (**j**) are the same. Daudi and Akata-BX1 loading controls in (**a**), (**c**), and (**e**) are the same, respectively. SNT16 loading controls in (**a**) and **Fig. S5a** are the same. TRL1 loading controls in (**c**), (**e**), and **Fig. S5a** are the same.

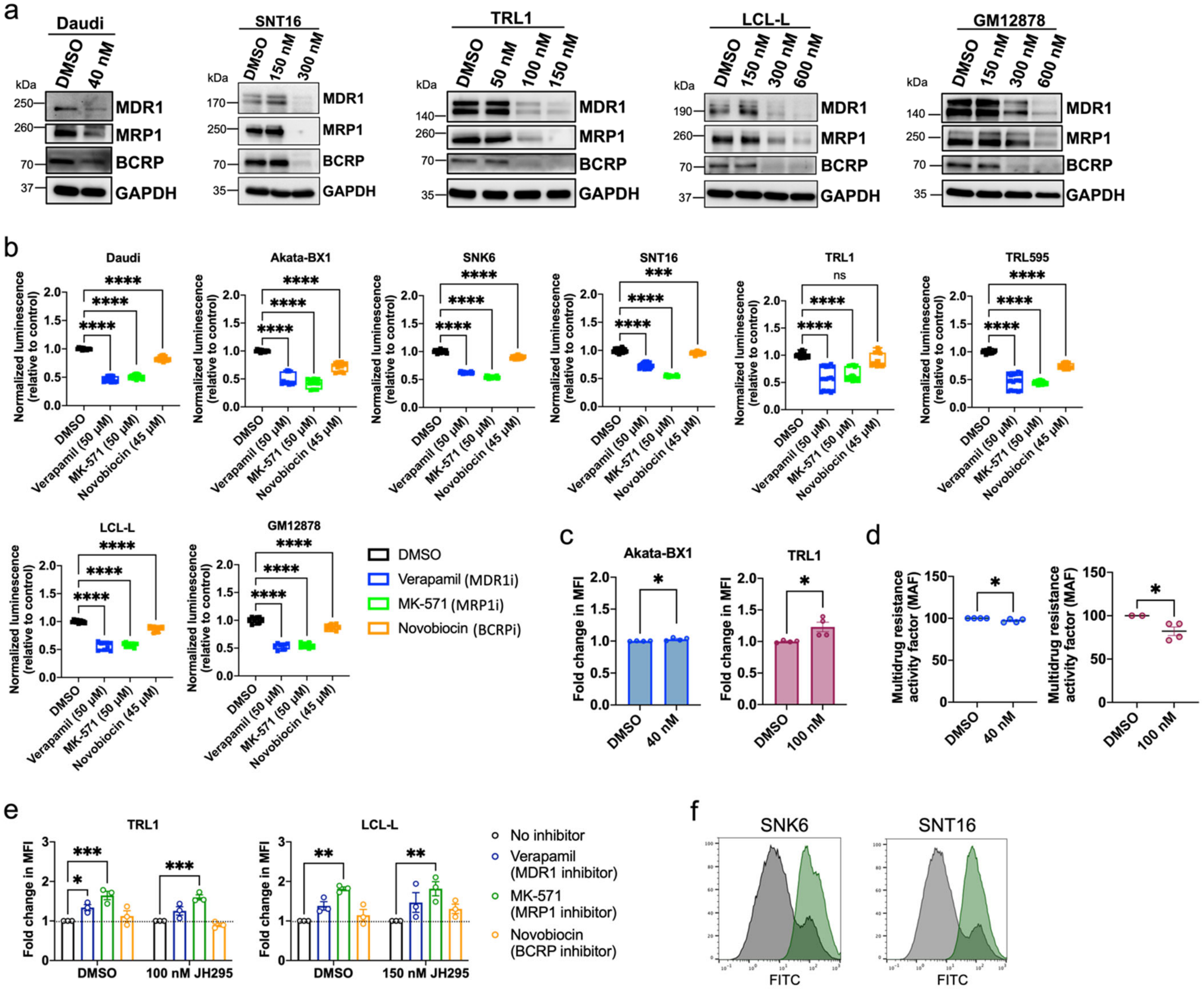

Fig. S5. NEK2 inhibition decreases expression and activity of ABC transporter proteins in EBV-positive NHL. (**a**) Western blot of MDR1, MRP1, and BCRP in EBV-positive NHL treated with either DMSO or increasing concentrations of JH295 for 48h. GAPDH was used as the loading control. Data are representative of N=3 biological replicates except for Daudi BCRP (N=1). The SNT16 western blot for MDR1 was stripped and reprobed with antibody against MRP1. (**b**) Cell viability assays of EBV-positive NHL treated with DMSO (control), verapamil (MDR1 inhibitor), MK-571 (MRP1 inhibitor), or novobiocin (BCRP inhibitor) for 48h. Data were normalized to the 0h luminescence values for each condition. N=3 biological replicates performed in a minimum of triplicate. Data were analyzed using one-way ANOVA with Dunnett's multiple comparisons test and graphed min to max. ****p<0.0001;

***p=0.0001; ns=not significant. (**c**) Multidrug resistance assays measuring ABC transporter protein activity in Akata-BX1 and TRL1 cells treated with either DMSO or JH295 for 48h. Following JH295 treatment, cells were loaded with the fluorescent substrate, $DiOC_2(3)$ (Akata-BX1; 20 minute loading period), or rhodamine (TRL1; 60 minute loading period), and the amount of substrate remaining within the cells after a 1.5-2h efflux period was measured by flow cytometry. Data are graphed as fold change in mean fluorescence intensity (MFI) relative to the DMSO control. N=2 biological replicates. Data were analyzed using t-test and graphed as mean ± SEM. *p=0.0209 Akata-BX1; *p=0.0111 TRL1. (**d**) Multidrug resistance factor (MAF) calculations using the data in (**c**). Data are normalized to the DMSO control, which was set at a MAF of 100. N=2 biological replicates. Data were analyzed using t-test and graphed as mean ± SEM. *p=0.0164 Akata-BX1; *p=0.0370 TLR1. (**e**) eFLUXX-ID® Green assays measuring activity of each individual ABC transporter protein in EBV-positive NHL treated ± JH295 for 48h. Data are graphed as fold change in MFI relative to the no inhibitor control for each condition. Dotted line represents no change (y=1). N=3 biological replicates. Data were analyzed using two-way ANOVA with Dunnett's multiple comparisons test and graphed as mean ± SEM. ***p<0.0005; **p≤0.005; *p=0.0251. (**f**) Histogram plot showing same data in **Fig. 4d** (SNK6 and SNT16) for the DMSO-treated cells (gray; control) and MK-571-treated cells (green; MRP1 inhibition). FITC intensity is plotted on the x-axis and increases moving toward the right. The more fluorescent substrate retained within the cells, the less ABC transporter protein activity. Plots are representative of N=3 biological replicates. Y-axes are normalized to mode. Events were gated on single-cell and PI-negative populations. SNT16 loading controls in (**a**) and **Fig. S4a** are the same. TRL1 loading controls in (**a**), **Fig. S4c**, and **Fig. S4e** are the same.

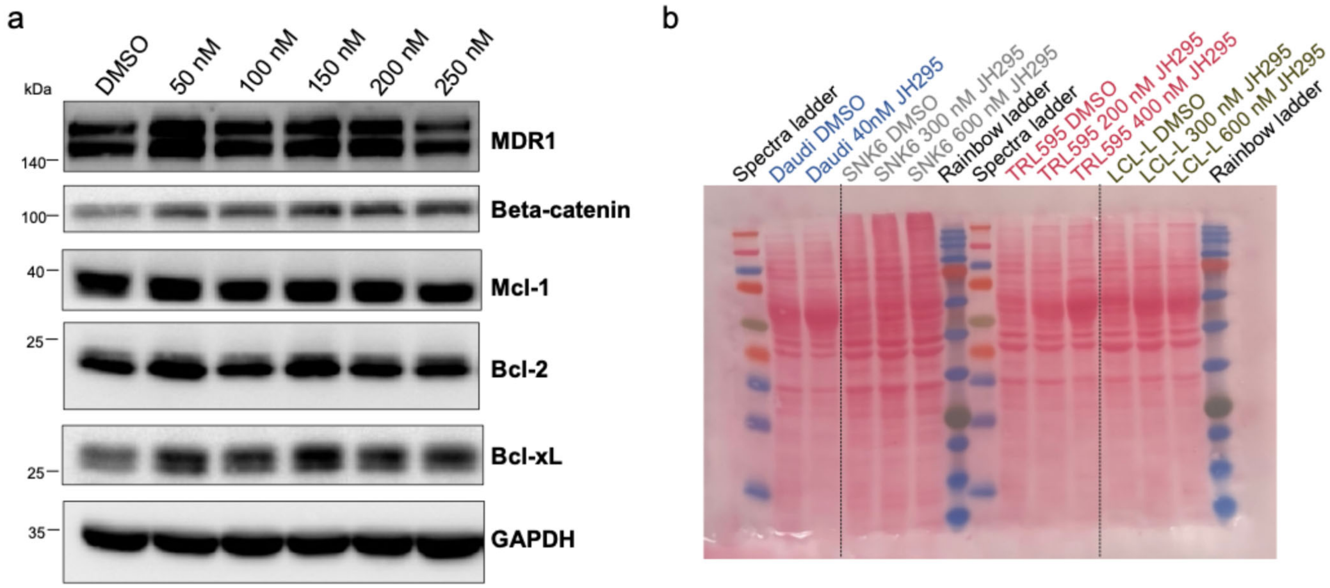

Fig. S6. JH295 treatment does not affect protein expression in PBMCs and does not induce global protein downregulation in EBV-positive NHL. (**a**) Western blot of PBMCs treated with DMSO or increasing concentrations of JH295 for 48h. GAPDH was used as the loading control. Data are representative of N=2 biological replicates from two unique cell donors. The western blot for Bcl-2 was stripped and reprobed with antibody against Bcl-xL. (**b**) Ponceau S protein staining of an immunoblot containing clarified protein lysates (50 μg protein/lane) from EBV-positive NHL cells treated with DMSO or increasing concentrations of JH295 for 48h. The blot was then processed for EBNA1 and GAPDH protein expression as shown in **Fig. S4k**. Spectra protein ladder=ThermoFisher; Rainbow protein ladder=Bioss Antibodies.

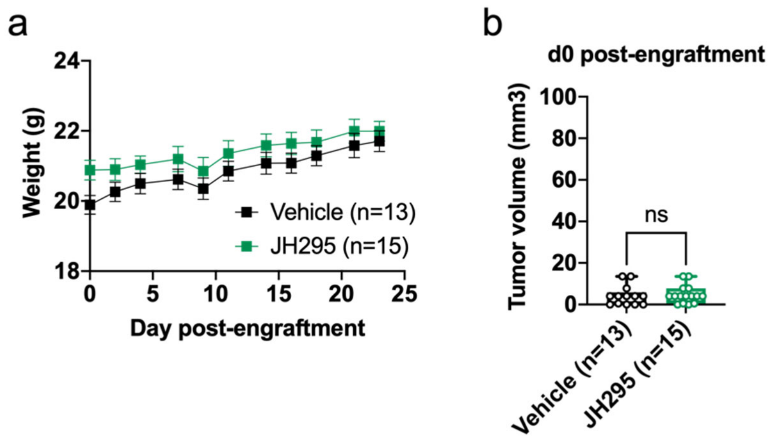

Fig. S7. JH295 reduces tumor burden in a SNK6 xenograft mouse model. (**a**) Weights of mice treated with DMSO (vehicle) or 15 mg/kg JH295. N=3 biological replicates. (**b**) SNK6 tumor volume in mice treated with DMSO or 15 mg/kg JH295 at day 0 post-engraftment. N=3 biological replicates. Data are plotted min to max and analyzed using Mann-Whitney test. ns=not significant.

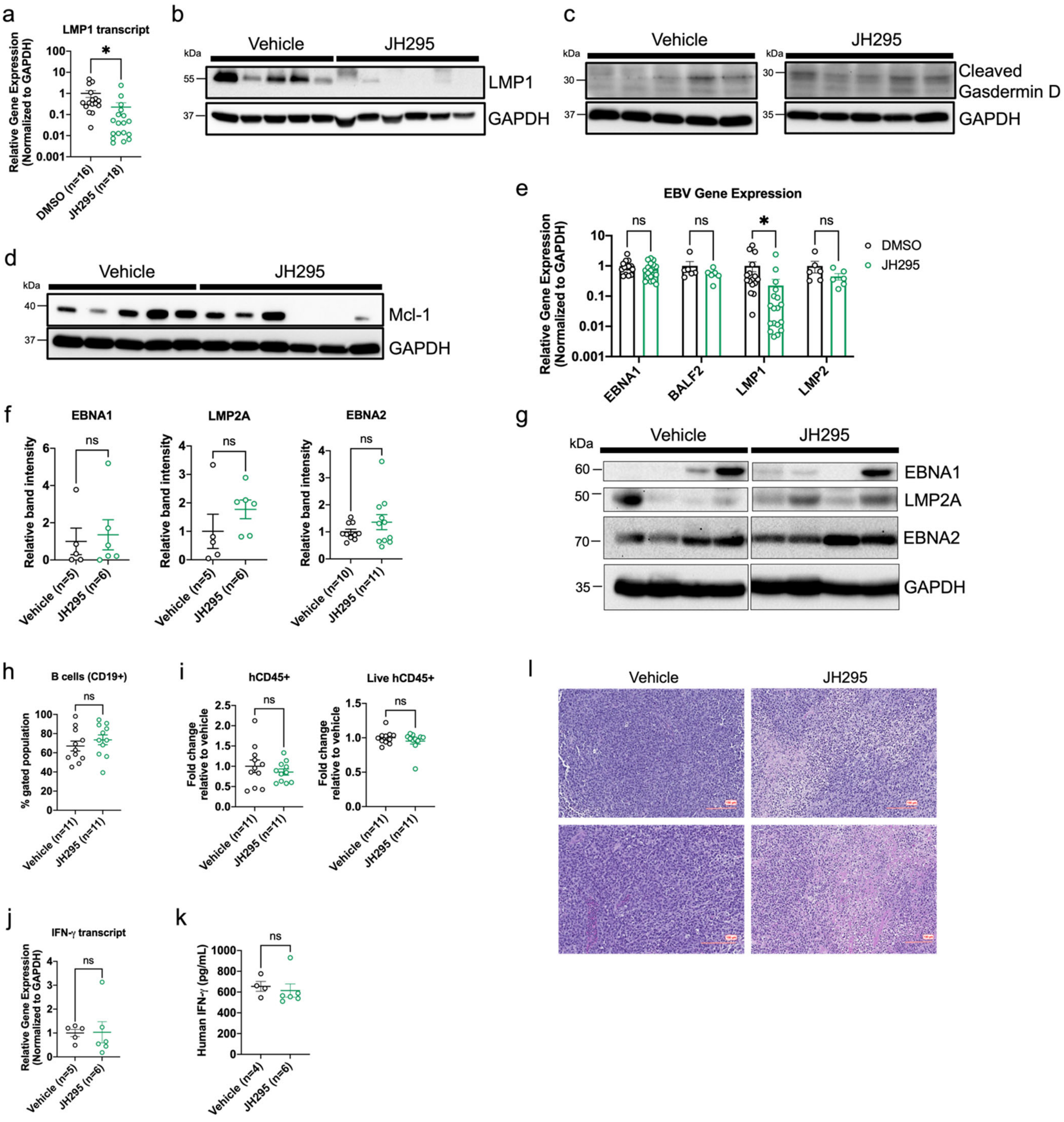

Fig. S8. JH295 treatment reduces expression of cellular and viral pro-survival proteins and facilitates tumor clearance in a cord blood-humanized mouse model of EBV-driven lymphomagenesis. (**a**) Quantification of LMP1 transcripts in the spleens of mice treated with either DMSO (black; N=16) or 15 mg/kg JH295 (green; N=18). N=3 biological replicates. Data were analyzed using t-test and graphed as mean ± SEM. *p=0.0207. (**b-d**) Western blots of LMP1 (**b**), cleaved

gasdermin D (**c**), and Mcl-1 (**d**) protein expression in the spleens of mice treated with either DMSO or 15 mg/kg JH295. GAPDH was used as the loading control. Data are representative of N=3 biological replicates. (**e**) Quantification of EBNA1, BALF2, LMP1, and LMP2 transcripts in the spleens of mice treated with either DMSO (black; N=6-16) or 15 mg/kg JH295 (green; N=6-18). Data were analyzed using two-way ANOVA with Sidak's multiple comparisons test and graphed as mean ± SEM. *p=0.0252; ns=not significant. The LMP1 transcript data in (**a**) and (**e**) are the same data set. (**f**) Quantification of EBNA1, LMP2A, and EBNA2 protein expression in the spleens of mice treated with either DMSO (black; N=5-10) or 15 mg/kg JH295 (green; N=6-11). Data were normalized to the GAPDH loading control. Data were analyzed using t-test and graphed as mean ± SEM. ns=not significant. (**g**) Western blot of EBNA1, LMP2A, and EBNA2 protein expression in the spleens of mice treated with either DMSO or 15 mg/kg JH295. GAPDH was used as the loading control. Data are representative of N=5-11 mice per treatment group. The western blot for LMP2A was stripped and reprobed with antibody against EBNA1. (**h**) Percentage of B cells (defined as CD19-positive) in the spleens of mice treated with either DMSO (black; N=11) or 15 mg/kg JH295 (green; N=11). Data are plotted as mean ± SEM and analyzed using t-test. ns=not significant. (**i**) Human CD45-positive and live human CD45-positive cells in the spleens of mice treated with either DMSO (black; N=11) or 15 mg/kg JH295 (green; N=11), normalized to the DMSO control. Data were analyzed using t-test and plotted as mean ± SEM. ns=not significant. (**j**) Quantification of interferon gamma transcripts in the spleens of mice treated with either DMSO (black; N=5) or 15 mg/kg JH295 (green; N=6). Data were analyzed using t-test and graphed as mean ± SEM. ns=not significant. (**k**) Quantification of interferon gamma in sera collected at necropsy from mice treated with either DMSO (black; N=4) or 15 mg/kg JH295 (green; N=6). Data were analyzed using t-test and graphed as mean ± SEM. ns=not significant. (**l**) Fixed tumor sections stained with hematoxylin

and eosin generated from mice treated with either DMSO or 15 mg/kg JH295. Images are representative of N=3 mice per treatment group. Scale bar=130 μm.

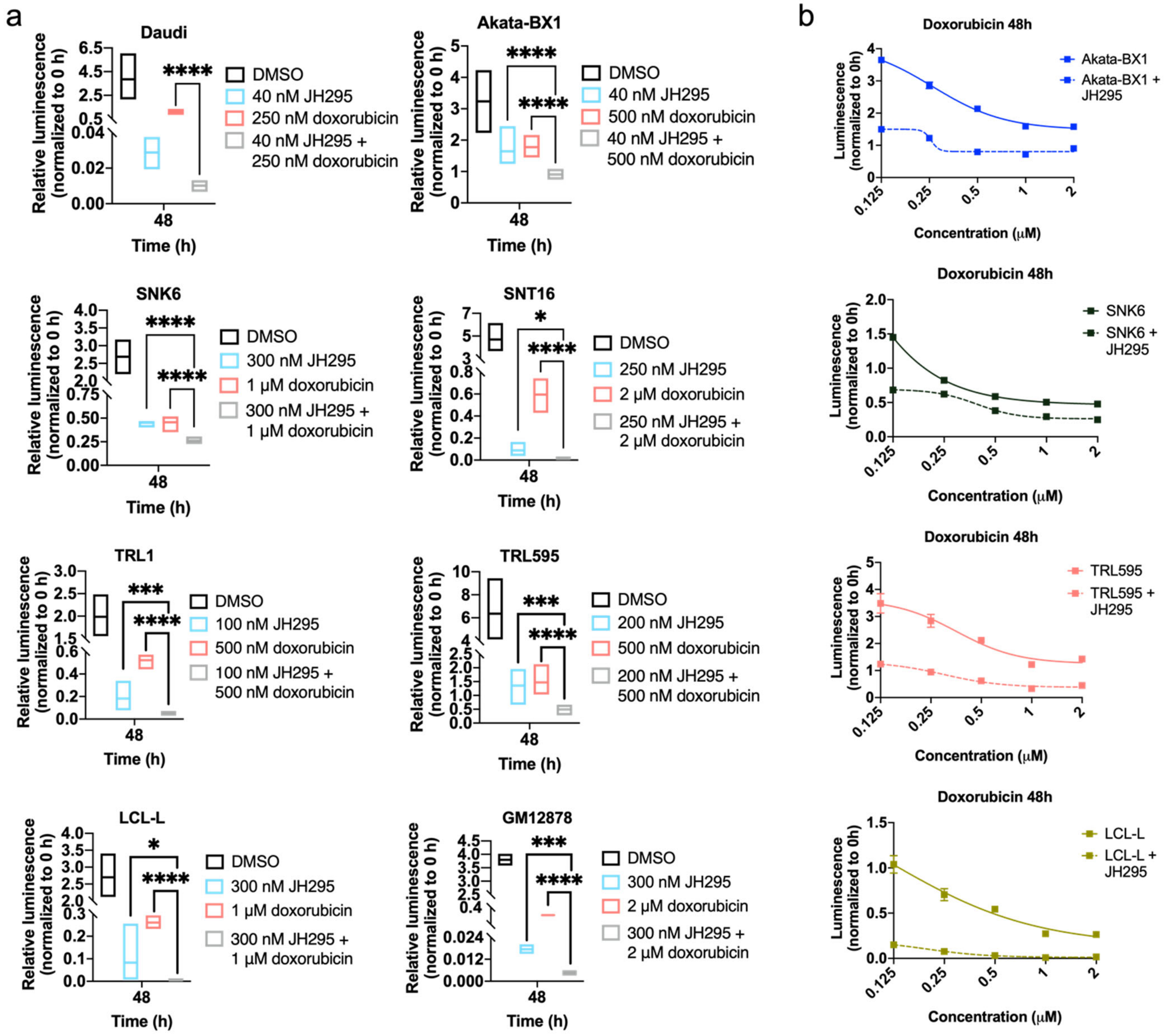

Fig. S9. JH295 treatment sensitizes EBV-positive NHL to doxorubicin. (**a**) Cell viability assays of EBV-positive NHL treated with DMSO (black bars), JH295 (aquamarine bars), doxorubicin (salmon bars), or JH295 + doxorubicin (gray bars) for 48h. Data were normalized to the 0h luminescence values for each condition. N=3 biological replicates performed in triplicate except SNK6 (N=2). For GM12878, only one replicate is graphed (representative of N=3 biological replicates); all others are pooled. Data were analyzed using one-way ANOVA with Holm-Sidak's multiple comparisons test and graphed min to max with line at mean. ****$p<0.0001$; ***$p<0.0005$; *$p=0.0473$ SNT16; *$p=0.0115$ LCL-L. (**b**) IC50 curves of EBV-positive NHL treated with increasing concentrations of doxorubicin in the presence or

absence of JH295 (same concentrations as in [**a**]). Curves were fit using non-linear regression and data are graphed as mean ± SEM. Data were normalized to the 0h luminescence values for each condition. Data are representative of N=3 biological replicates performed in triplicate.

Table S1. Characteristics of EBV-positive lymphoma cell lines.

Cell line	*Latency stage*	*Disease*
Daudi	I	Burkitt lymphoma
Akata-BX1	I	Burkitt lymphoma
SNK6	II	NK/T cell lymphoma
SNT16	II	NK/T cell lymphoma
TRL1	III	Post-transplant lymphoma
TRL595	III	Post-transplant lymphoma
LCL-L	III	EBV-transformed primary B cells
GM12878	III	EBV-transformed primary B cells

Table S2. JH295 GI50 values for EBV-positive lymphoma cell lines.

Cell line	*JH295 GI50 (48h)*
Daudi	0.036 μM
Akata-BX1	0.052 μM
SNK6	0.190 μM
SNT16	0.158 μM
TRL1	0.065 μM
TRL595	0.139 μM
LCL-L	0.163 μM
GM12878	0.130 μM

Table S3. Comparison of JH295 and NBI-961 GI50 values for EBV-positive lymphoma cell lines.

Cell line	*JH295 GI50 (48h)*	*NBI-961 GI50 (48h)*
Akata-BX1	0.052 μM	0.097 μM
SNK6	0.190 μM	0.077 μM
TRL595	0.139 μM	0.153 μM
LCL-L	0.163 μM	0.104 μM

SI References

1. M. C. White, J. P. Wong, B. Damania, Inhibition of NEK2 Promotes Chemosensitivity and Reduces KSHV-positive Primary Effusion Lymphoma Burden. *Cancer Res Commun* **4**, 1024-1040 (2024).
